# Supplementary figures and images for: Spliced Leader Trapping Reveals Widespread Alternative Splicing Patterns in the Highly Dynamic Transcriptome of Trypanosoma brucei
Source: PLoS Pathog. 2010 Aug 5;6(8):e1001037. doi: 10.1371/journal.ppat.1001037 (PMC2916883; doi:10.1371/journal.ppat.1001037)

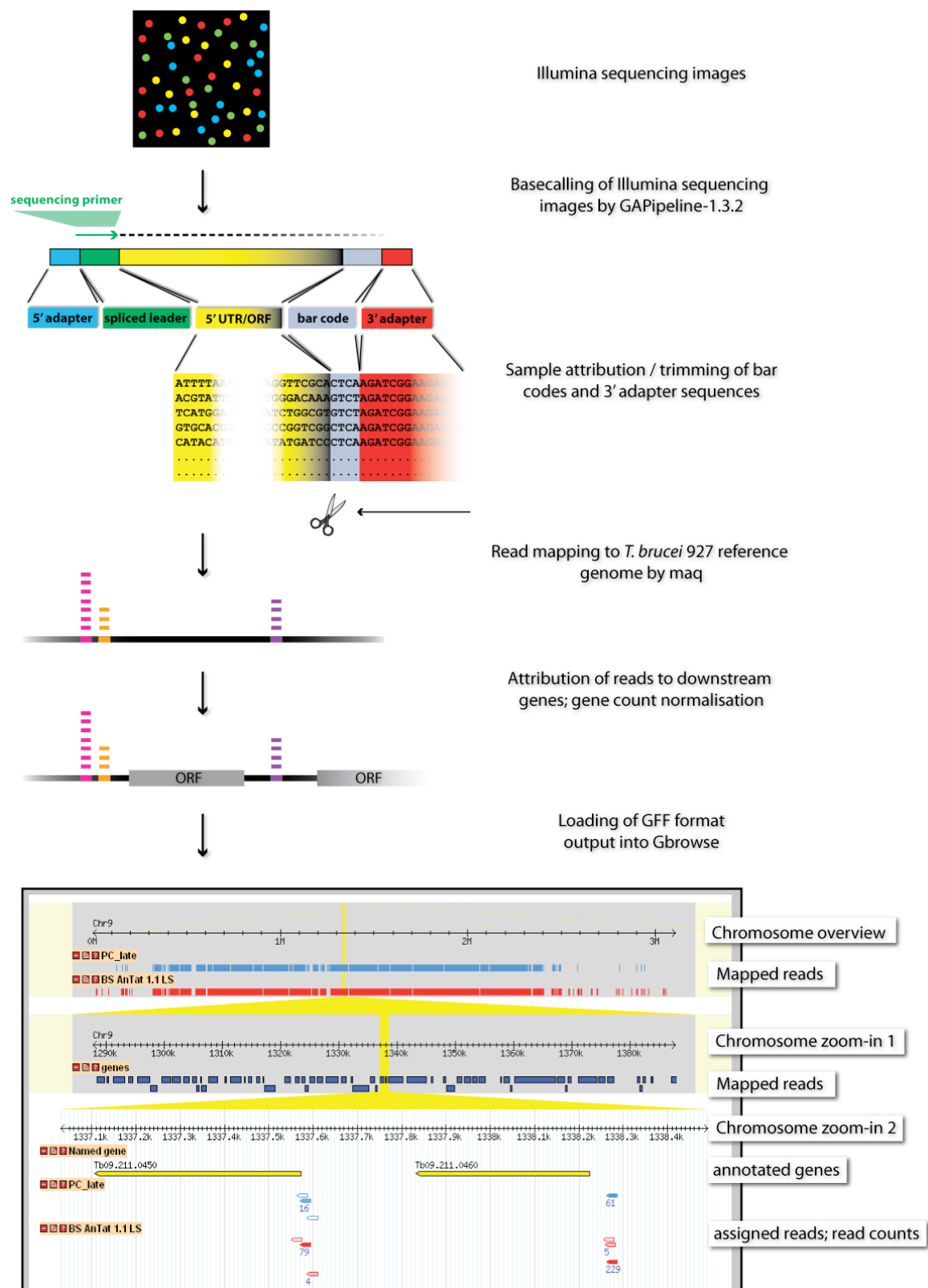

B

Figure S1

Supplement: Figure S1 — Spliced leader trapping approach. Schematic description of (A) library preparation, sequencing and (B) bioinformatics analysis. (0.62 MB PDF) [file ppat.1001037.s001.pdf]

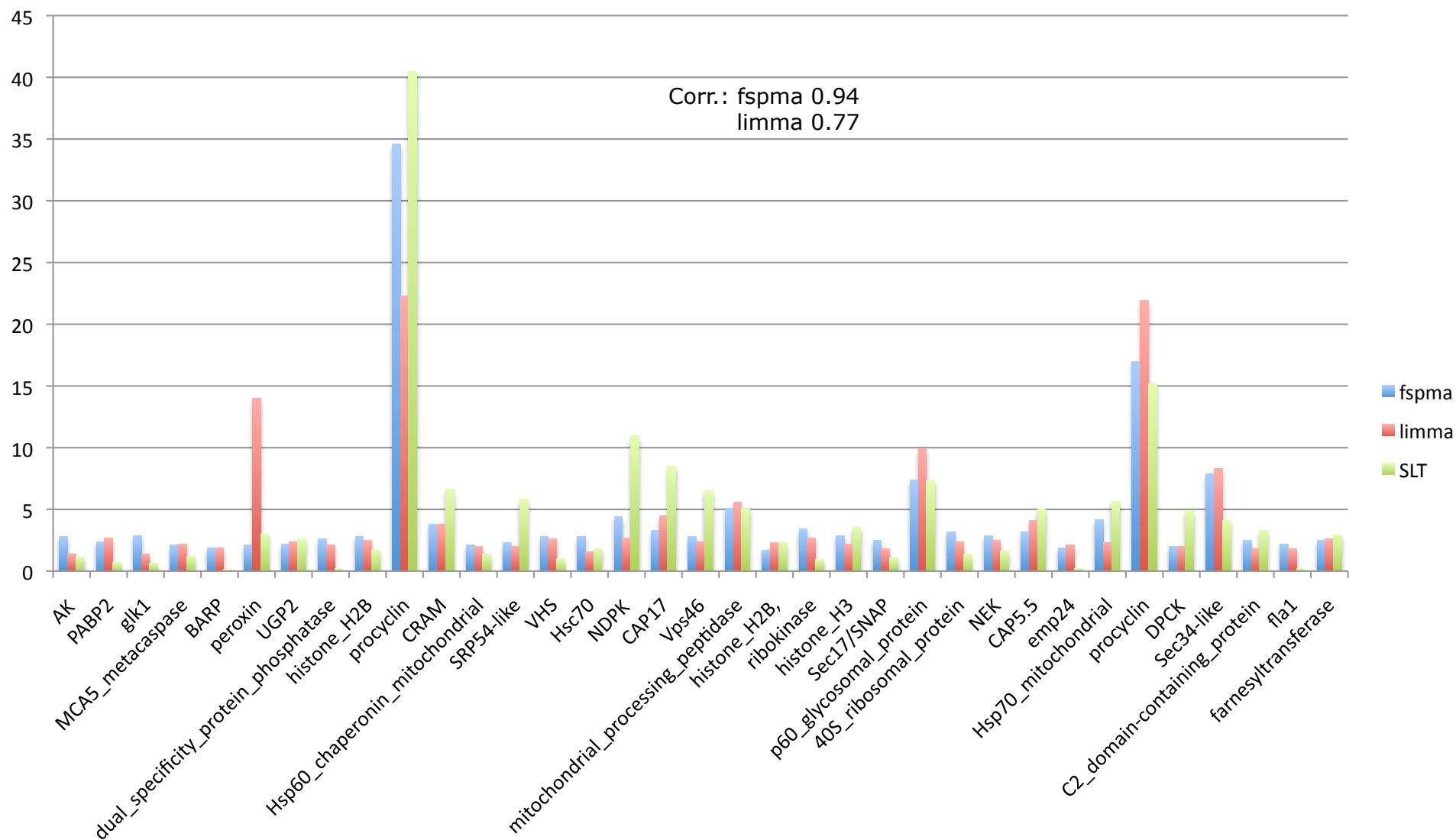

Figure S2

Supplement: Figure S2 — Microarray correlation. Correlation between the SLT approach and a recent microarray study by Koumandou et al. (2008). Transcripts more abundant in procyclic forms than in the bloodstream forms correlated with a coefficient of 0.77 or 0.93 to the SLT approach depending on the statistics (fspma or limma) used in the study by Koumandou and coworkers [11]. (0.66 MB PDF) [file ppat.1001037.s002.pdf]

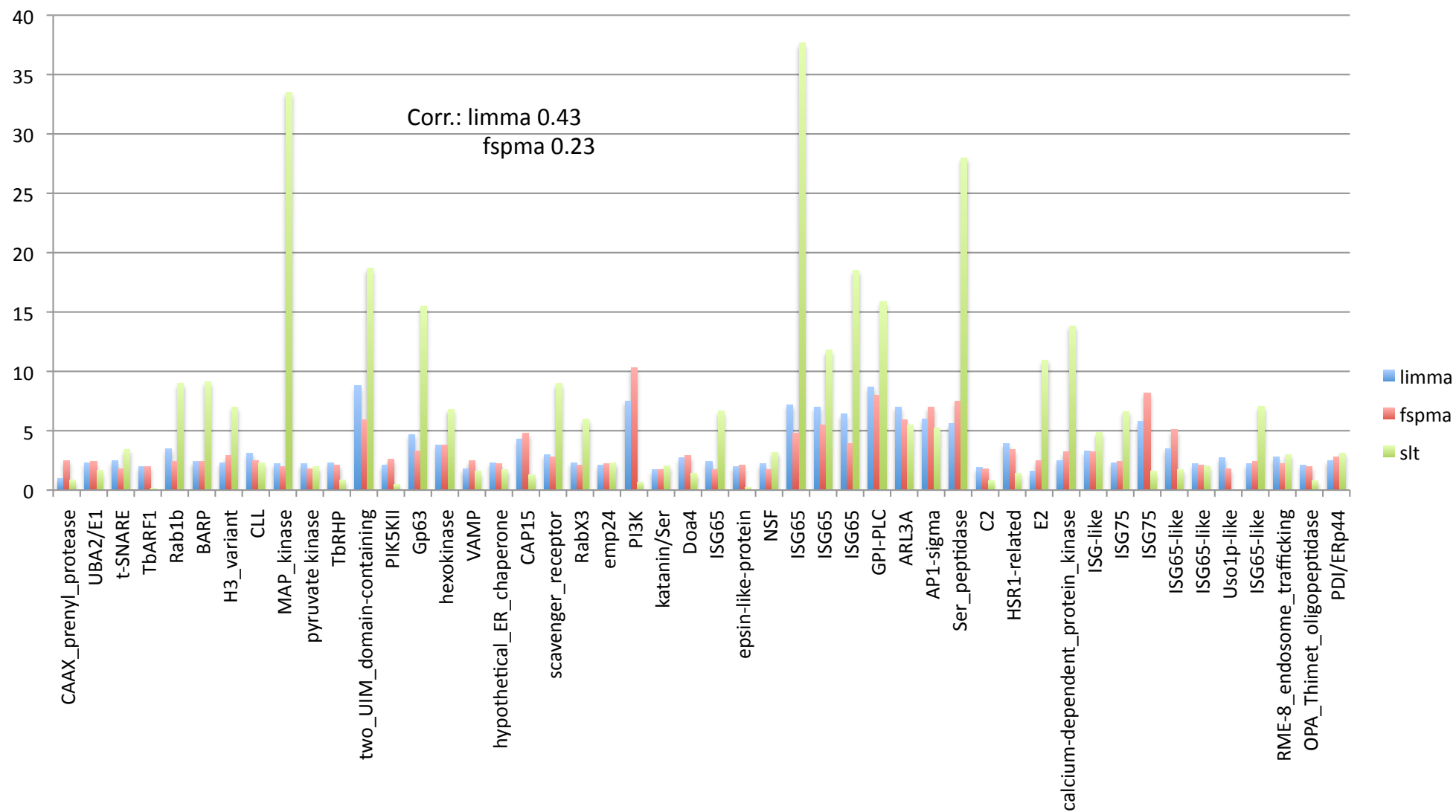

Figure S3

Supplement: Figure S3 — Microarray correlation. Correlation between the SLT approach and a recent microarray study by Koumandou et al. (2008). Transcripts more abundant in bloodstream forms than the procyclic forms correlated with a coefficient of 0.43 or 0.23 to the SLT approach depending on the statistics (fspma or limma) used in the study by Koumandou and coworkers [11]. (0.38 MB PDF) [file ppat.1001037.s003.pdf]

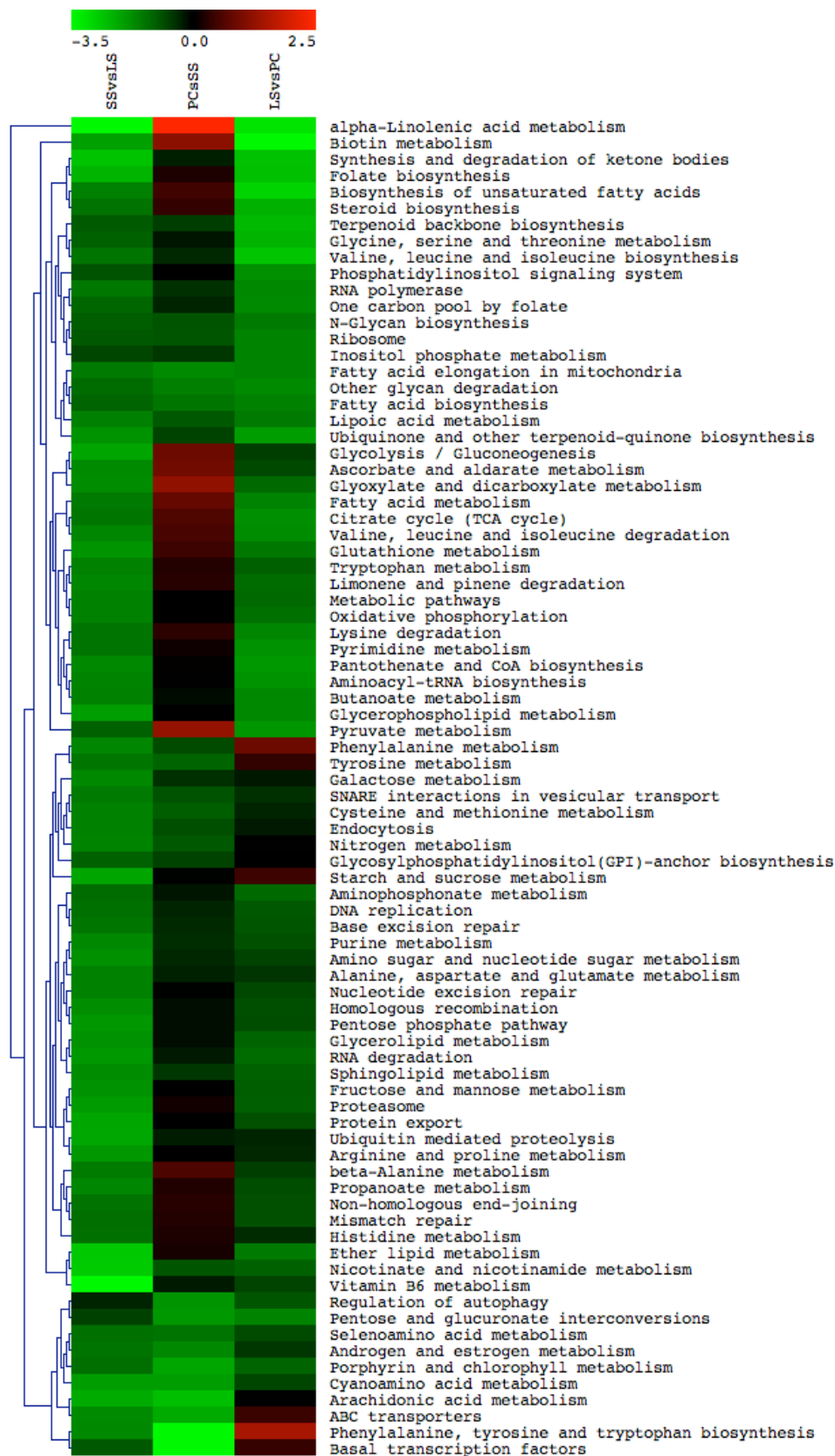

Figure S4

Supplement: Figure S4 — Expression profile of KEEG pathways. Heatmap of log2 changes for KEGG pathway genes for three life cycle stages of T. brucei. The dendrogram was obtained using hierarchical average linkage clustering of euclidian distances. LS, long slender bloodstream form; SS: short stumpy bloodstream form; PC, procyclic form. (0.18 MB PDF) [file ppat.1001037.s004.pdf]

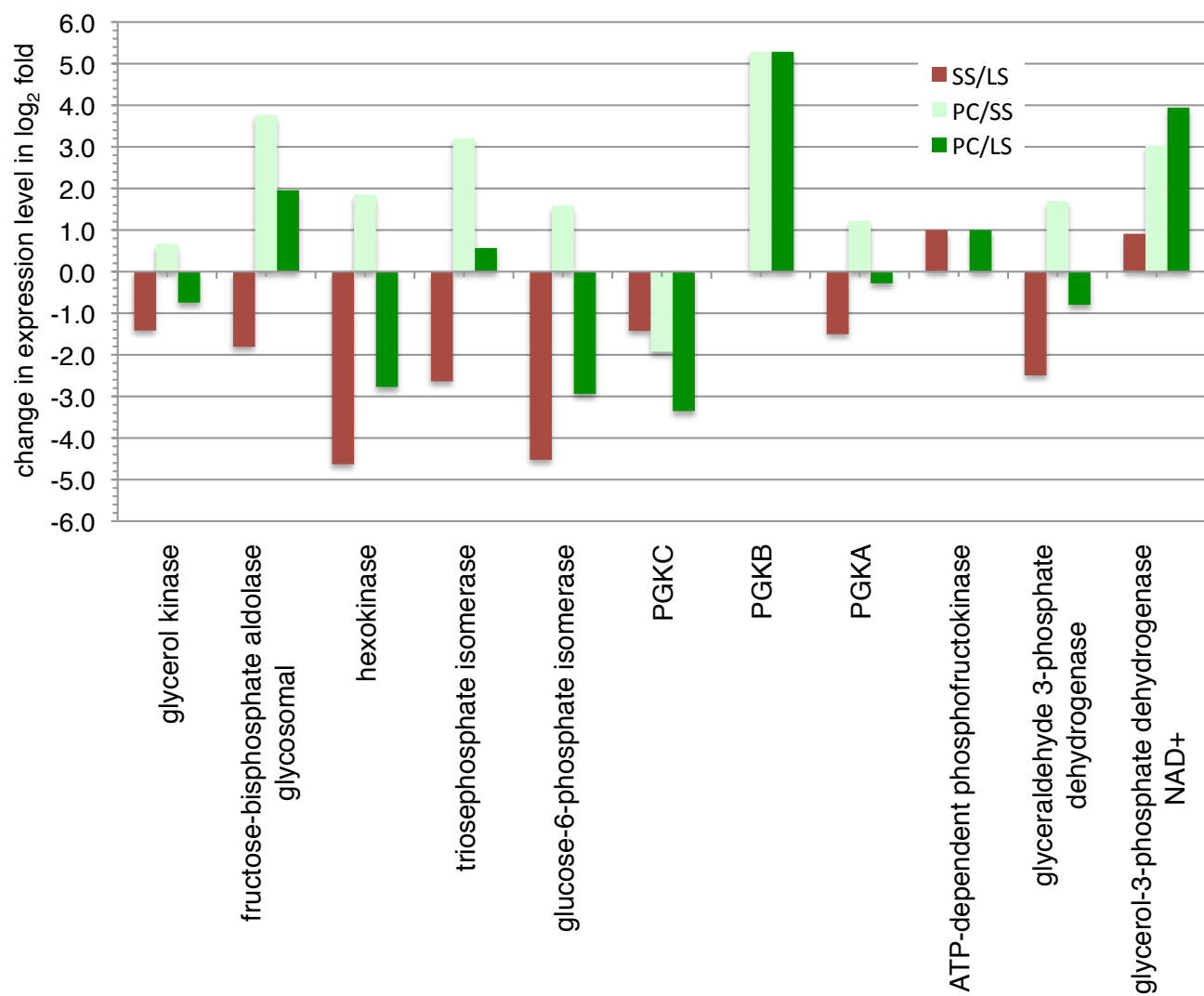

Figure S5

Supplement: Figure S5 — Expression profile glycolysis pathway. Regulation of the glycolytic pathway as annotated in KEGG. The differences in expression between long slender bloodstream and short stumpy (maroon), short stumpy and procyclic (light green) and long slender bloodstream from and procyclic form (dark green) are shown in log2 fold. (0.09 MB PDF) [file ppat.1001037.s005.pdf]

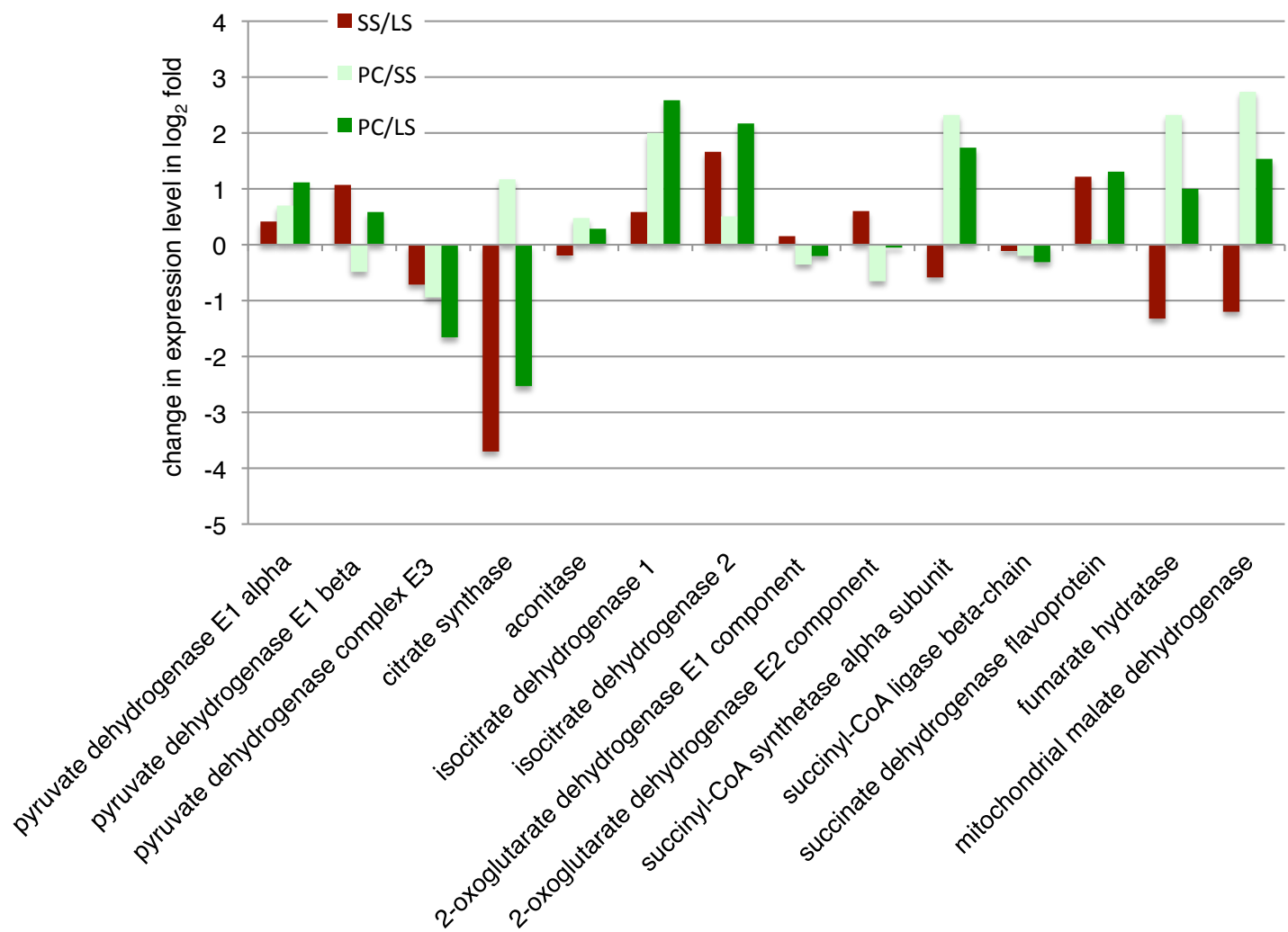

Figure S6

Supplement: Figure S6 — Expression profile oxidative phosphorylation pathway. The regulation of the citric acid cycle pathway as annotated in KEGG. The differences in expression between long slender bloodstream and short stumpy (maroon), short stumpy and procyclic (light green) and long slender bloodstream form and procyclic form (dark green) are shown in log2 fold. (0.26 MB PDF) [file ppat.1001037.s006.pdf]

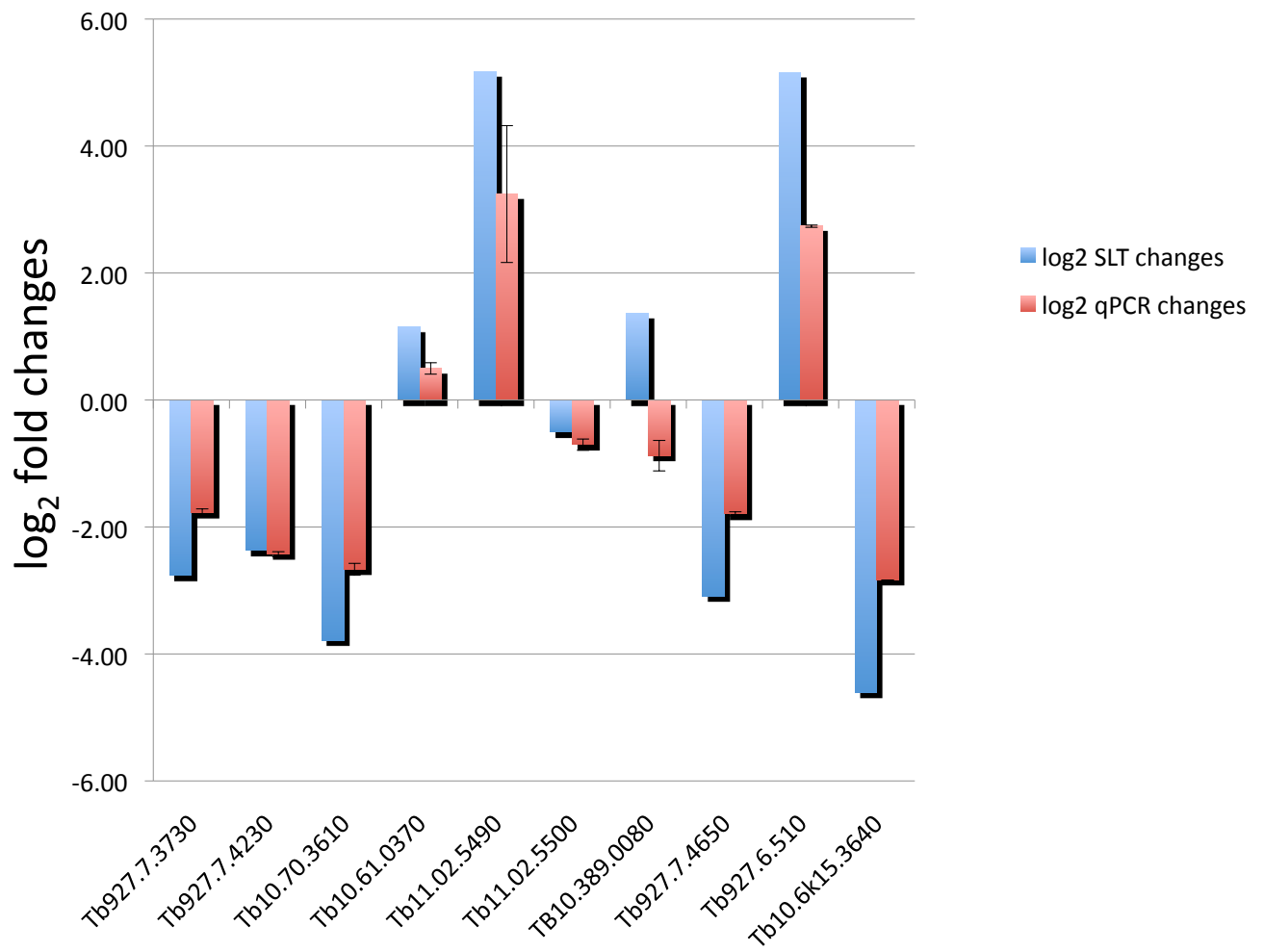

Figure S7

Supplement: Figure S7 — Correlation RT qPCR and SLT. Comparison of expression levels between SLT and RT qPCR from procyclic over long slender bloodstream form cells. Log2 fold changes of expression are shown for the SLT (blue) and RT qPCR. A positive value indicates higher steady state level RNA abundance for the corresponding gene in procyclic cells. (0.17 MB PDF) [file ppat.1001037.s007.pdf]

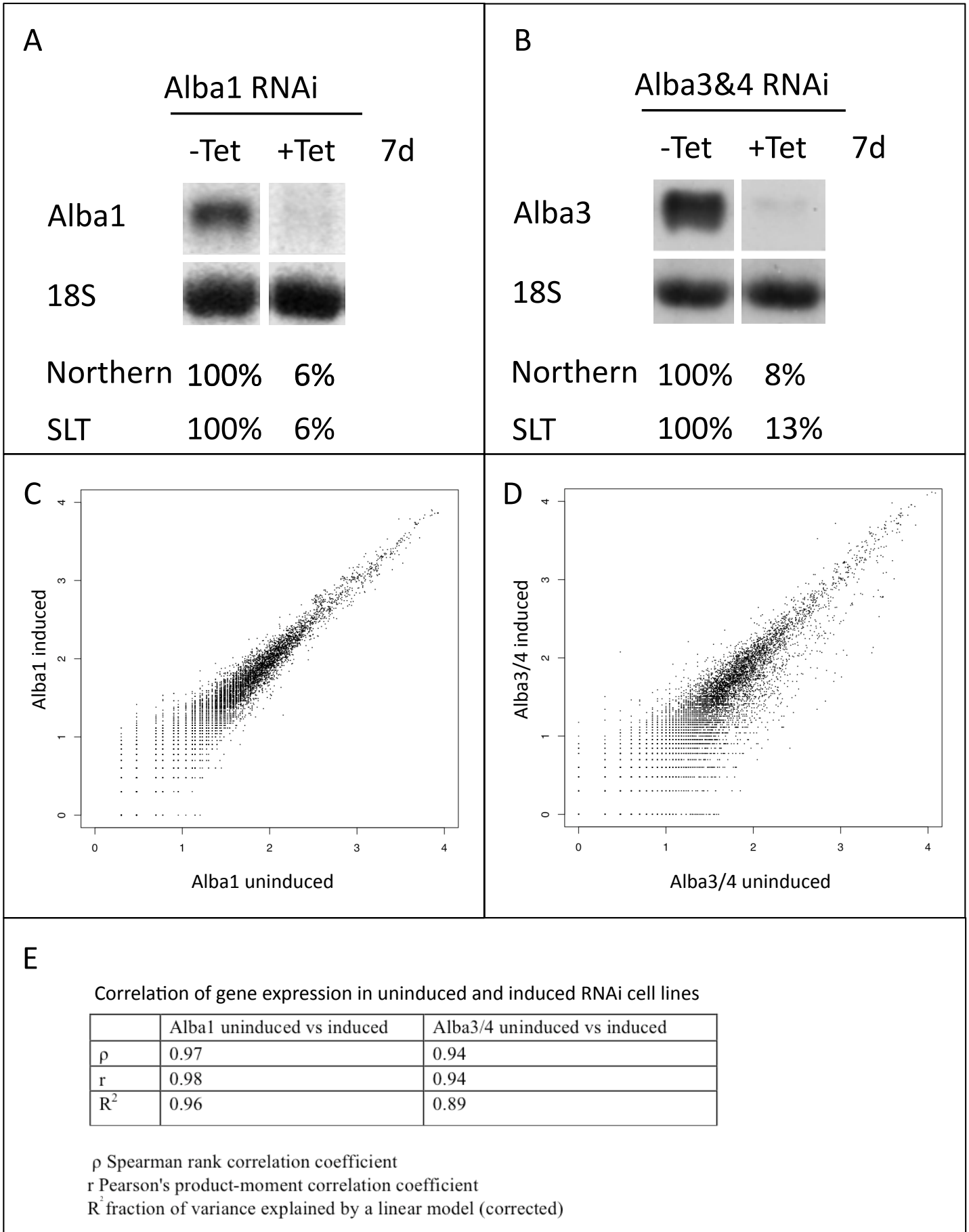

Figure S8

Supplement: Figure S8 — Comparison RNAi libraries uninduced induced. Comparison of SLT and Northern hybridization. (A and B) Relative levels of RNA transcripts detected by either SLT or Northern blot analysis using RNAi cell lines Alba1 and Alba 3/4. −Tet/+Tet refers to RNA isolated from uninduced and induced cell lines after 7 days. Quantitation of Northern blots was performed with a Phosphoimager using the 18S rRNA as a loading control. RNA levels from uninduced cells were set at 100%. (C and D) Scatter plots of mRNA expression levels (log10 tags per million) between two libraries. Each dot represents one gene. (C) RNAi experiment Alba1_N non-induced vs. Alba1_I induced. (D) RNAi experiment Alba34_N non-induced vs. Alba34_I induced. (E) Overall correlation of steady state RNA levels of the entire genome from uninduced and induced cell lines. (0.27 MB PDF) [file ppat.1001037.s008.pdf]

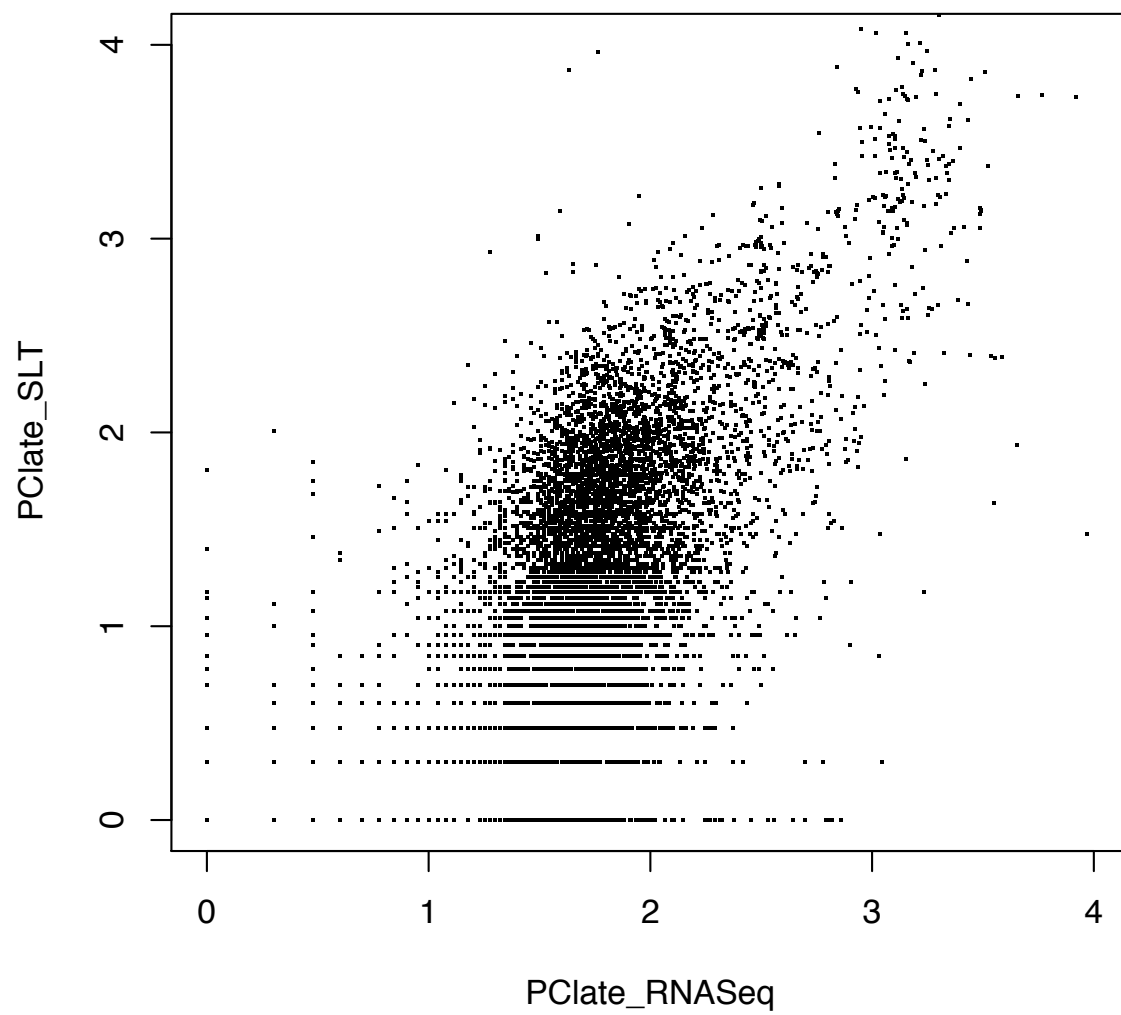

Figure S9

Supplement: Figure S9 — Scatter plot expression profile comparison SLT and RNA seq. Scatter plot of mRNA expression levels (log10 tags per million) between SLT and RNAseq from poly(A) procyclic T. brucei mRNA. (0.09 MB PDF) [file ppat.1001037.s009.pdf]

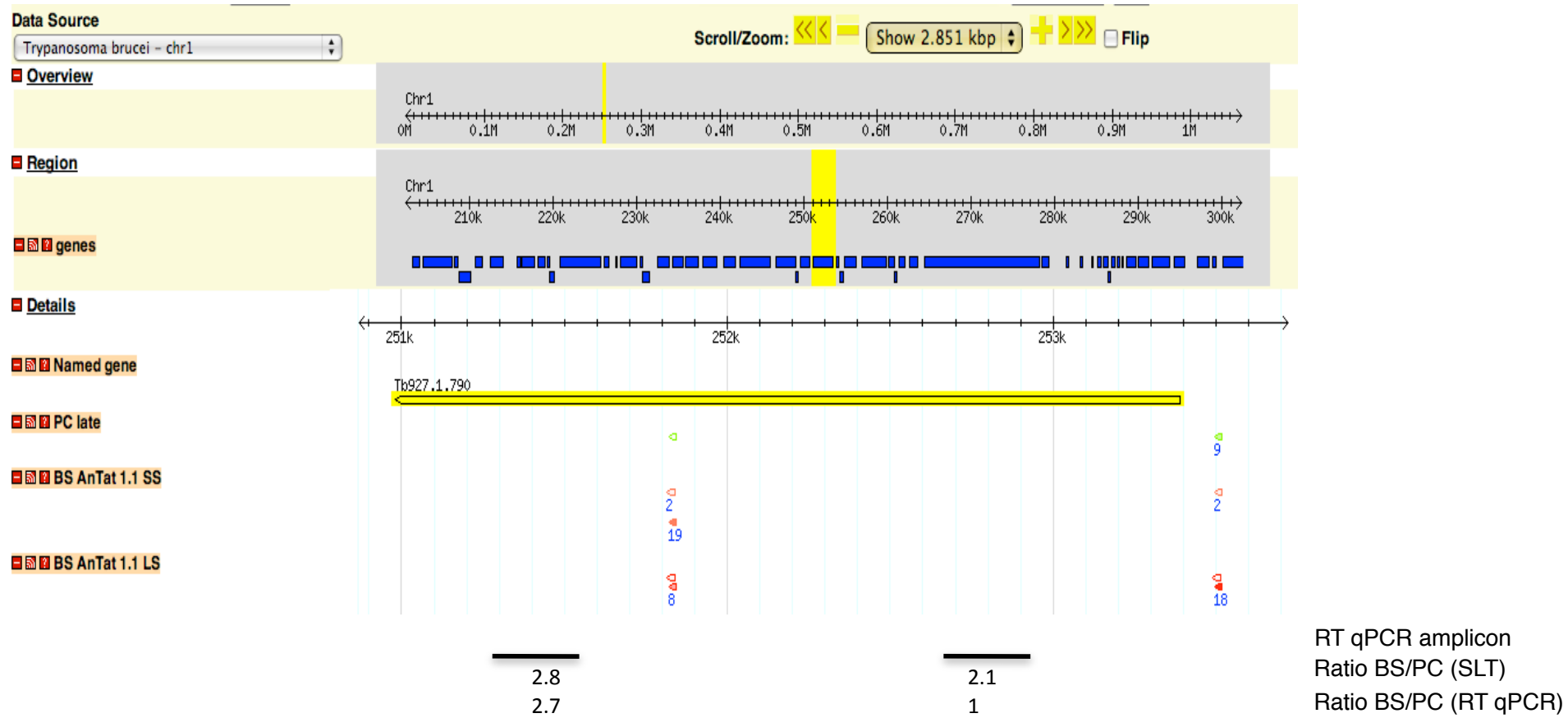

Figure S10

Supplement: Figure S10 — Alternative splice variant A. Example of splicing type A, no downstream AUG is found in the reading frame of the annotated gene. This also represents an example of a differentially spliced transcript, where the major splice site changes between long slender (LS), short stumpy (SS) and procyclic form (PC). RT qPCR amplicon shows the region that was used for RT qPCR. Ratio BS/PC (SLT) indicates the ratio of SLT tags, for the downstream tags the upstream tags are added in order to be comparable to the qPCR results (i.e. 1 downstream+9 upstream). (0.07 MB PDF) [file ppat.1001037.s010.pdf]

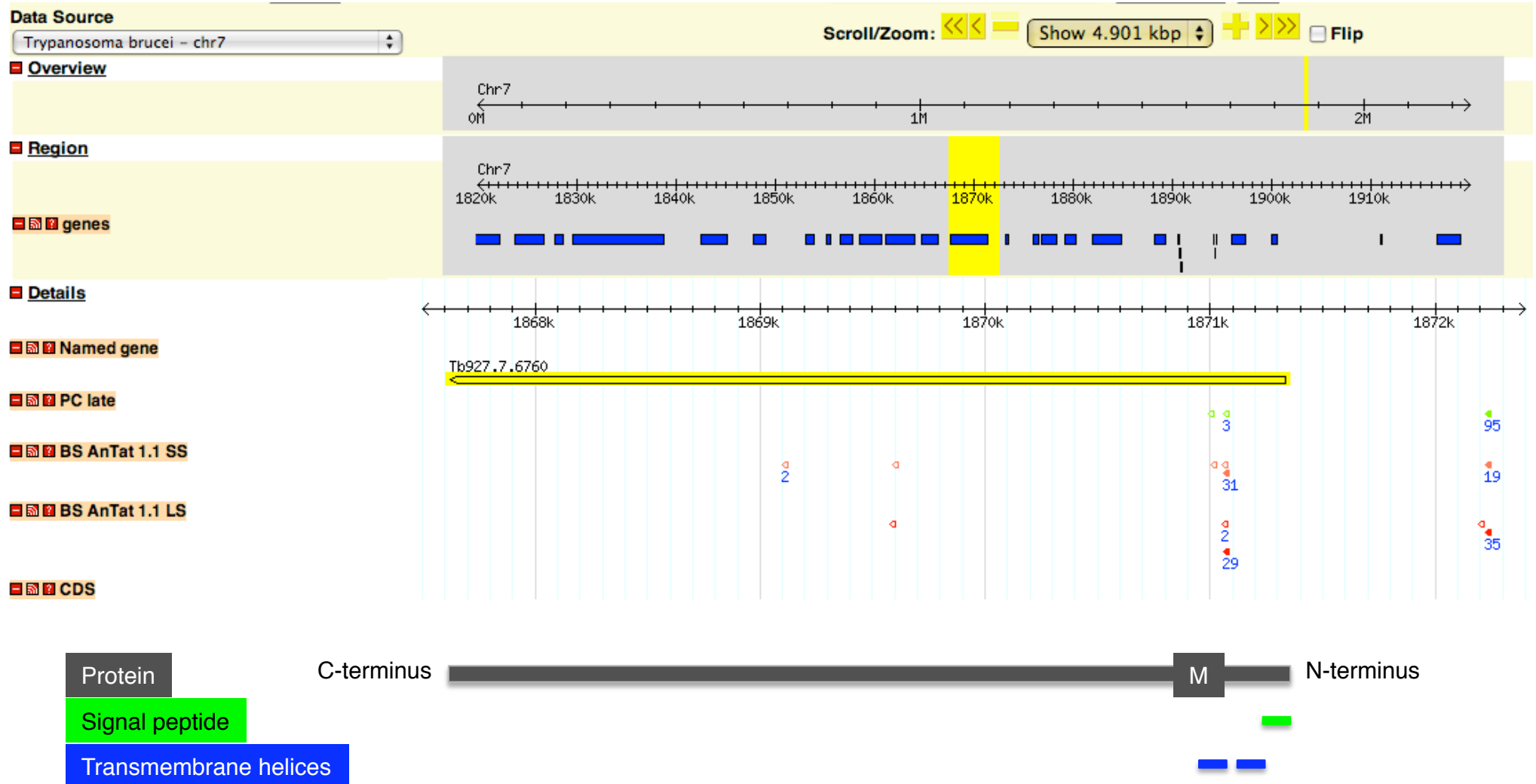

Figure S11

Supplement: Figure S11 — Aternative splice variant B. Example of splicing type B with a downstream AUG (M) in the reading frame of the annotated gene, but use of that AUG would lead to loss of the signal peptide predicted by SingalP. This also represents an example of a differentially spliced transcript where the major splice site changes between the long slender (LS), short stumpy (SS) and the procyclic form (PC). (0.08 MB PDF) [file ppat.1001037.s011.pdf]

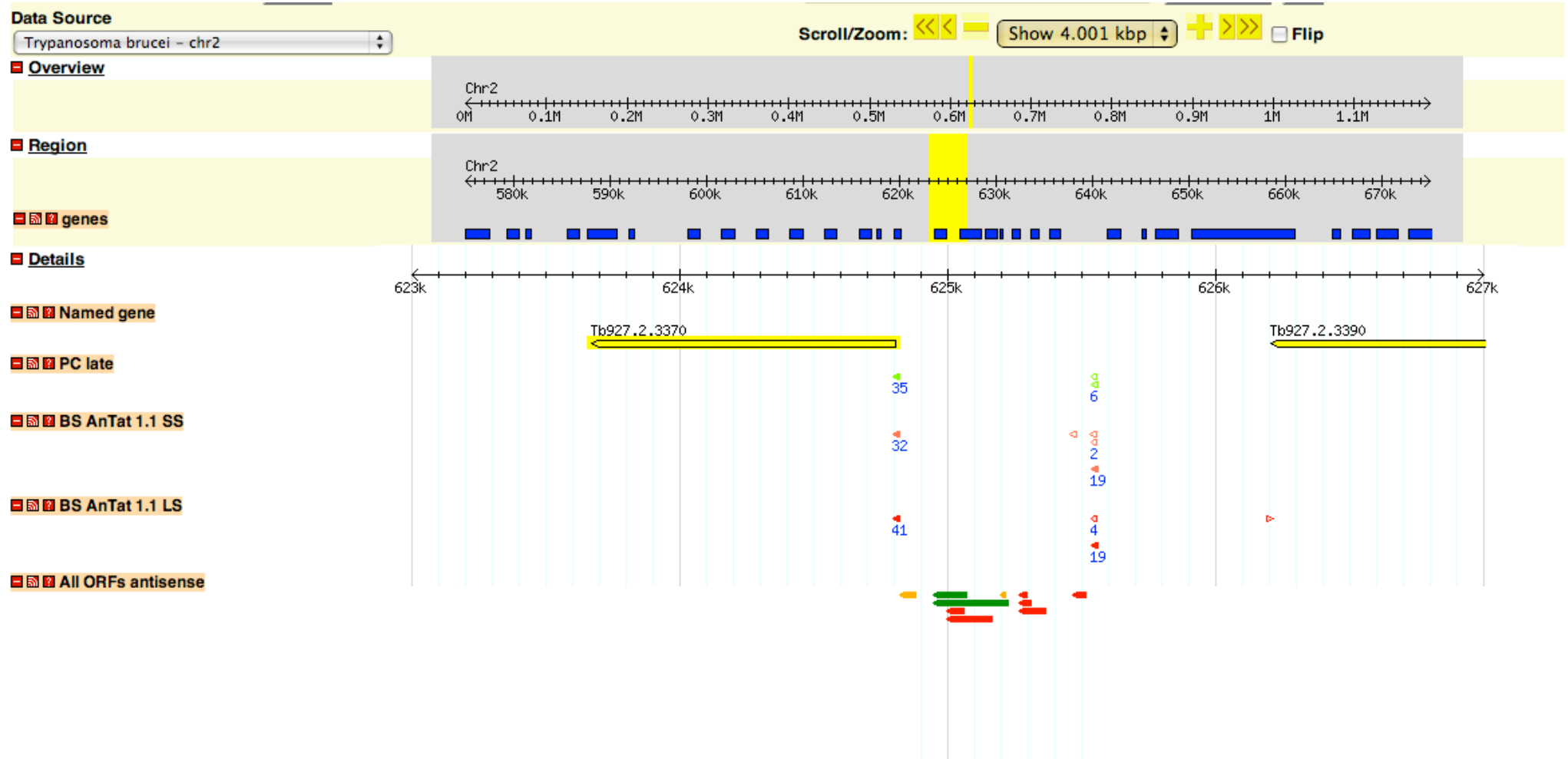

Figure S12

Supplement: Figure S12 — Alternative splice variant C. Example of splicing type C with several uORFs between the two alternative splice sites. uORFs are in color according to the reading frame. The minimum length for an uORF was set to six amino acids. Long slender (LS), short stumpy (SS) and the procyclic form (PC). (0.08 MB PDF) [file ppat.1001037.s012.pdf]

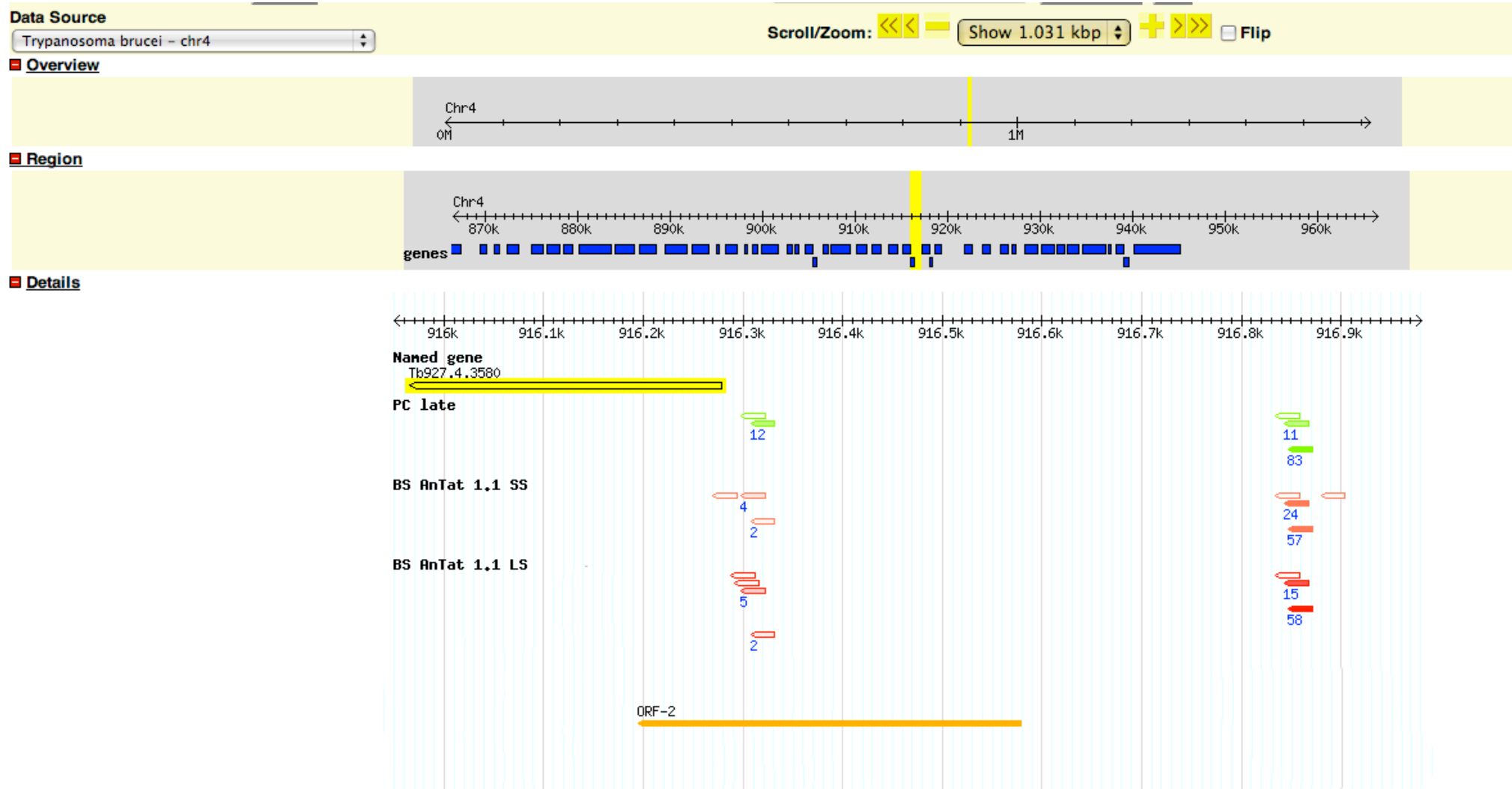

Figure S13

Supplement: Figure S13 — Alternative splice variant D. Example of splicing type D with an overlapping open reading frame of 384 bases (ORF2). Long slender (LS), short stumpy (SS) and procyclic form (PC). (0.09 MB PDF) [file ppat.1001037.s013.pdf]
