# Supplementary material for: Spliced Leader Trapping Reveals Widespread Alternative Splicing Patterns in the Highly Dynamic Transcriptome of Trypanosoma brucei
Source: PLoS Pathog. 2010 Aug 5;6(8):e1001037. doi: 10.1371/journal.ppat.1001037 (PMC2916883; doi:10.1371/journal.ppat.1001037)
Supplement: Table S1 — Correlation of SLT expression profile (0.04 MB PDF) [file ppat.1001037.s014.pdf]

Table S1: Expression level correlation of SLT approach with previous studies

Gene ID(s)	Name/putative function	Stage up	Comment	References	Fold change
Tb10.6k15.3640	Alternative oxidase	BSF	Northern	Chaudhuri et al. J Eukaryot Microbiol. 2002 Jul-Aug;49(4):263-9	23
Tb927.4.4730	Amino acid transporter	PROC	Northern (data not shown)	Robles & Clayton Mol Biochem Parasitol. 2008 Jan;157(1):102-6	16
Tb927.4.3950	CAP5.5/ TbCALP4	PROC	qPCR	Olego-Fernandez et al. Protist. 2009 Aug 3. [Epub ahead of print]	5
Tb927.8.8330	CAP5.5V /TbCALP8	BSF	qPCR	Olego-Fernandez et al. Protist. 2009 Aug 3. [Epub ahead of print]	15
Tb10.70.0830	Clathrin heavy chain;TbCLH	BSF	Northern	Morgan et al. J Cell Sci. 2001 Jul;114(Pt 14):2605-1	10
Tb10.6k15.3510	CRAM	PROC	Northern	Lee et al. Mol Cell Biol. 1990 Sep;10(9):4506-17	7
Tb927.8.1890	Cytochrome C1	PROC	Northern, protein data	Torri, A.F. and Hajduk, S.L. (1988) Posttranscriptional regulation of cytochrome c expression during the devel-opmental cycle of Trypanosoma brucei. Mol. Cell. Biol. 8, 4625-4633.	4
Tb10.6k15.2180	cytochrome oxidase IX	PROC	Northern	Mayho et al. Nucleic Acids Res. 2006;34(18):5312-24. Epub 2006 Sep 29	3
Tb09.160.1820	cytochrome oxidase V	PROC	Northern	Mayho et al. Nucleic Acids Res. 2006;34(18):5312-24. Epub 2006 Sep 29	31
Tb10.100.0160	cytochrome oxidase VI	PROC	Northern	Mayho et al. Nucleic Acids Res. 2006;34(18):5312-24. Epub 2006 Sep 29	8
Tb927.4.4620	cytochrome oxidase VIII	PROC	Northern	Mayho et al. Nucleic Acids Res. 2006;34(18):5312-24. Epub 2006 Sep 29	16
Tb11.01.4702	cytochrome oxidase X	PROC	Northern	Mayho et al. Nucleic Acids Res. 2006;34(18):5312-24. Epub 2006 Sep 29	3
Tb927.2.4210	Glycosomal PEPCK	PROC	protein data for differential expression	Kueng et al. J Biol Chem. 1989 Mar 25;264(9):5203-9	7
Tb927.2.6000	GPI-phospholipase	BSF	Northern	Carrington et al. Mol Biochem Parasitol. 1989 Mar 15;33(3):289-96.	14
Tb927.2.3270; Tb927.2.3280; Tb927.2.3290; Tb927.2.3300; Tb927.2.3310; Tb927.2.3320	ISG65	BSF	Northern	Ziegelbauer et al. J Biol Chem. 1992 May 25;267(15):10797-803	28
Tb09.211.4511; Tb09.211.4512; Tb09.211.4513	KMP-11 (basal body protein)	PROC	protein data for differential expression	Stebeck et al. Mol Biochem Parasitol. 1995 Apr;71(1):1-1	4
Tb10.70.5110	malate dehydrogenase	PROC	Northern	Anderson et al. Exp Parasitol. 1998 May;89(1):63-70	3
Tb927.1.710	PGKB	PROC	Northern	Colasante et al. Mol Biochem Parasitol. 2007 Feb;151(2):193-204.	20
Tb927.1.700	PGKC	BSF	Northern	Colasante et al. Mol Biochem Parasitol. 2007 Feb;151(2):193-204.	9
Tb927.6.520; Tb927.6.510; Tb927.6.480; Tb927.6.450; Tb10.6k15.0030; Tb10.6k15.0020	PROCYCLINS	PROC	Northern; qPCR	Roditi et al. Nature. 1987 Jan 15-21;325(6101):272-4. Urwyler et al. PLoS Pathog. 2005 Nov;1(3):e22. Epub 2005 Nov 4	27
