# Supplementary material for: Spliced Leader Trapping Reveals Widespread Alternative Splicing Patterns in the Highly Dynamic Transcriptome of Trypanosoma brucei
Source: PLoS Pathog. 2010 Aug 5;6(8):e1001037. doi: 10.1371/journal.ppat.1001037 (PMC2916883; doi:10.1371/journal.ppat.1001037)
Supplement: Table S2 — Robustly regulated transcripts (0.08 MB PDF) [file ppat.1001037.s015.pdf]

Table S2: Robustly regulated transcripts

ID	Long slender/ procyclics	Product	EC
Tb927.2.1700	up	hypothetical protein, conserved	
Tb927.2.1920	up	expression site-associated gene (ESAG) protein, putative,expression site-associated gene 5 (ESAG5) protein, putative	
Tb927.2.2020	up	expression site-associated gene (ESAG) protein, putative,expression site-associated gene 3 (ESAG3) protein, putative	
Tb927.2.2060	up	variant surface glycoprotein (VSG)-related, putative	
Tb927.2.2070	up	hypothetical protein	
Tb927.2.2120	up	protein kinase, putative,NIMA/Nek Serine/threonine-protein kinase family, putative	
Tb927.2.3270	up	65 kDa invariant surface glycoprotein	
Tb927.2.3300	up	65 kDa invariant surface glycoprotein	
Tb927.2.3320	up	65 kDa invariant surface glycoprotein	
Tb927.2.3340	up	hypothetical protein	
Tb927.2.4670	up	hypothetical protein, conserved	
Tb927.2.6000	up	glycosylphosphatidylinositol-specific phospholipase C,VSG lipase	EC:4.6.1.14
Tb927.2.6180	up	iron/ascorbate oxidoreductase family protein, putative	
Tb927.2.6200	up	adenosine transporter 2, putative	
Tb927.2.6230	up	iron/ascorbate oxidoreductase family protein, putative	
Tb927.2.6240	up	adenosine transporter 2	
Tb927.2.6280	up	adenosine transporter 2, putative	
Tb927.2.6310	up	iron/ascorbate oxidoreductase family protein, putative	
Tb927.2.6320	up	adenosine transporter 2, putative	
Tb927.3.560	up	expression site-associated gene (ESAG) protein, putative,expression site-associated gene 11 (ESAG11) protein, putative	
Tb927.3.570	up	expression site-associated gene (ESAG) protein, putative,expression site-associated gene 2 (ESAG2) protein, putative	
Tb927.3.580	up	leucine-rich repeat protein (LRRP), putative	
Tb927.3.600	up	hypothetical protein	
Tb927.3.1470	up	variant surface glycoprotein (VSG)-related, putative	
Tb927.3.1480	up	hypothetical protein, conserved	
Tb927.3.1490	up	leucine-rich repeat protein (LRRP), putative	
Tb927.3.1500	up	variant surface glycoprotein (VSG)-related, putative	
Tb927.3.1510	up	variant surface glycoprotein (VSG)-related, putative	
Tb927.3.1660	up	hypothetical protein, conserved	
Tb927.3.2510	up	expression site-associated gene (ESAG) protein, putative,expression site-associated gene 2 (ESAG2) protein, putative	

Table S2: Robustly regulated transcripts

Tb927.3.2540	up	variant surface glycoprotein (VSG)-related, putative	
Tb927.3.2590	up	hypothetical protein	
Tb927.3.2610	up	hypothetical protein, conserved	
Tb927.3.3380	up	TFIIF-stimulated CTD phosphatase, putative	
Tb927.3.3870	up	lipase domain protein, putative	
Tb927.3.4180	up	hypothetical protein	
Tb927.3.4230	up	subtilisin-like serine peptidase,serine peptidase, clan SB, family S8-like protein	
Tb927.3.5080	up	hypothetical protein, conserved	
Tb927.3.5660	up	UDP-Gal or UDP-GlcNAc-dependent glycosyltransferase, putative	
Tb927.3.5670	up	hypothetical protein	
Tb927.3.5680	up	variant surface glycoprotein (VSG)-related, putative	
Tb927.3.5690	up	hypothetical protein, conserved	
Tb927.4.500	up	hypothetical protein, conserved	
Tb927.4.710	up	hypothetical protein, conserved	
Tb927.4.810	up	expression site-associated gene (ESAG) protein, putative,expression site-associated gene 5 (ESAG5) protein, putative	
Tb927.4.1230	up	hypothetical protein	
Tb927.4.1640	up	UDP-galactose transporter, putative	
Tb927.4.2380	up	sarcoplasmic reticulum glycoprotein, putative	
Tb927.4.2760	up	hypothetical protein, conserved	
Tb927.4.3980	up	chaperone protein DNAJ, putative	
Tb927.4.4020	up	amino acid transporter, putative	
Tb927.4.4410	up	receptor-type adenylate cyclase GRESAG 4, putative	EC:4.6.1.1
Tb927.4.4430	up	receptor-type adenylate cyclase GRESAG 4, putative	EC:4.6.1.1
Tb927.4.4440	up	receptor-type adenylate cyclase GRESAG 4, putative	EC:4.6.1.1
Tb927.4.4460	up	receptor-type adenylate cyclase GRESAG 4, putative,receptor-type adenylate cyclase GRESAG 4.4B	EC:4.6.1.1
Tb927.4.4580	up	hypothetical protein, conserved	
Tb927.4.4900	up	hypothetical protein	
Tb927.4.5310	up	serine/threonine-protein kinase A, putative,protein kinase, putative	EC:2.7.1.37
Tb927.5.130	up	variant surface glycoprotein (VSG)-related, putative	
Tb927.5.140	up	hypothetical protein, conserved	
Tb927.5.310	up	hypothetical protein	
Tb927.5.350	up	75 kDa invariant surface glycoprotein, putative	
Tb927.5.370	up	75 kDa invariant surface glycoprotein, putative	
Tb927.5.390	up	75 kDa invariant surface glycoprotein, putative	
Tb927.5.400	up	75 kDa invariant surface glycoprotein, putative	

Table S2: Robustly regulated transcripts

Tb927.5.610	up	acidic phosphatase, putative	
Tb927.5.630	up	acidic phosphatase, putative	
Tb927.5.1380	up	hypothetical protein	
Tb927.5.1390	up	64 kDa invariant surface glycoprotein	
Tb927.5.1410	up	64 kDa invariant surface glycoprotein	
Tb927.5.1430	up	64 kDa invariant surface glycoprotei	
Tb927.5.1440	up	hypothetical protein	
Tb927.5.1970	up	hypothetical protein	
Tb927.5.1990	up	hypothetical protein, conserved	
Tb927.5.2000	up	hypothetical protein	
Tb927.5.2010	up	hypothetical protein	
Tb927.5.3220	up	signal peptidase type I, putative,serine peptidase, Clan SF, Family S26A	
Tb927.5.3600	up	ATP-dependent DEAD/H RNA helicase, putative	
Tb927.5.3660	up	hypothetical protein, conserved	
Tb927.5.4010	up	hypothetical protein	
Tb927.5.4600	up	expression site-associated gene (ESAG) protein, putative,expression site-associated gene 3 (ESAG3) protein, putative	
Tb927.5.4620	up	expression site-associated gene (ESAG) protein, putative,expression site-associated gene 9 (ESAG9) protein, putative	
Tb927.5.4630	up	expression site-associated gene (ESAG) protein, putative,expression site-associated gene 1 (ESAG1) protein, putative	
Tb927.6.350	up	hypothetical protein, conserved	
Tb927.6.440	up	haptoglobin-hemoglobin receptor	
Tb927.6.560	up	cysteine peptidase C (CPC),CPC cysteine peptidase, Clan CA, family C1, Cathepsin B-like	EC:3.4.22.2
Tb927.6.930	up	metacaspase MCA3,cysteine peptidase, Clan CD, family C13, putative	
Tb06.3A7.960	up	hypothetical protein	
Tb927.6.1310	up	hypothetical protein, conserved	
Tb927.6.1350	up	hypothetical protein, conserved	
Tb927.6.1460	up	cyclin 3,mitotic cyclin, putative	
Tb927.6.3000	up	fatty acid desaturase, putative,sphingolipid delta 4 desaturase, putative	
Tb927.6.3120	up	chaperone protein DNAJ, putative	
Tb927.6.3480	up	RNA-binding protein, putative	
Tb927.6.3640	up	hypothetical protein, conserved	
Tb927.6.5000	up	hypothetical protein, conserved	
Tb927.7.180	up	hypothetical protein	
Tb927.7.300	up	UDP-Gal or UDP-GlcNAc-dependent glycosyltransferase, putative	
Tb927.7.470	up	hypothetical protein	
Tb927.7.1420	up	hypothetical protein, conserved	
Tb927.7.3250	up	expression site-associated gene (ESAG) protein, putative,expression site-associated gene 6 (ESAG6) protein, putative	

Table S2: Robustly regulated transcripts

Tb927.7.3260	up	expression site-associated gene (ESAG) protein, putative,expression site-associated gene 7 (ESAG7) protein, putative	
Tb927.7.3720	up	hypothetical protein	
Tb927.7.3730	up	RNA-binding protein, putative	
Tb927.7.4110	up	kinesin, putative	
Tb927.7.4230	up	hypothetical protein, conserved	
Tb927.7.4260	up	hypothetical protein, conserved	
Tb927.7.4500	up	hypothetical protein, conserved	
Tb927.7.4650	up	SNF2 DNA repair protein, putative	
Tb927.7.4690	up	hypothetical protein	
Tb927.7.5830	up	hypothetical protein, conserved	
Tb927.7.5890	up	hypothetical protein, conserved	
Tb927.7.6570	up	hypothetical protein, conserved	
Tb927.7.6600	up	hypothetical protein, conserved	
Tb927.7.6860	up	expression site-associated gene (ESAG) protein, putative,expression site-associated gene 5 (ESAG5) protein, putative	
Tb927.7.7510	up	hypothetical protein	
Tb927.7.7520	up	receptor-type adenylate cyclase GRESAG 4, putative	EC:4.6.1.1
Tb927.7.7530	up	receptor-type adenylate cyclase GRESAG 4, putative	EC:4.6.1.1
Tb927.8.1200	up	vacuolar-type Ca2+-ATPase 2	EC:3.6.3.-
Tb927.8.2070	up	hypothetical protein, conserved	
Tb927.8.2080	up	hypothetical protein, conserved	
Tb927.8.2780	up	RNA-binding protein RBP10, putative	
Tb927.8.3390	up	hypothetical protein, conserved	
Tb927.8.3720	up	hypothetical protein, conserved	
Tb927.8.3730	up	hypothetical protein, conserved	
Tb927.8.4060	up	flagellum-adhesion glycoprotein, putative	
Tb927.8.4340	up	hypothetical protein, conserved	
Tb927.8.5050	up	hypothetical protein, conserved	
Tb927.8.5140	up	hypothetical protein, conserved	
Tb927.8.5870	up	hypothetical protein, conserved	
Tb927.8.5910	up	hypothetical protein	
Tb927.8.6710	up	hypothetical protein, conserved	
Tb927.8.6720	up	hypothetical protein, conserved	
Tb927.8.6730	up	hypothetical protein, conserved	
Tb927.8.7300	up	variant surface glycoprotein (VSG)-related, putative	
Tb927.8.7310	up	hypothetical protein	
Tb927.8.7330	up	hypothetical protein	
Tb927.8.7710	up	hypothetical protein, conserved	
Tb927.8.7760	up	hypothetical protein, conserved	
Tb927.8.7780	up	hypothetical protein, conserved	
Tb927.8.7820	up	hypothetical protein, conserved	
Tb927.8.7920	up	receptor-type adenylate cyclase GRESAG 4, putative	EC:4.6.1.1
Tb927.8.7970	up	hypothetical protein	
Tb927.8.8000	up	hypothetical protein, conserved	
Tb927.8.8270	up	hypothetical protein, conserved	
Tb927.8.8320	up	hypothetical protein, conserved	

Table S2: Robustly regulated transcripts

Tb927.8.8330	up	calpain, putative,cysteine peptidase, putative	
Tb09.142.0310	up	expression site-associated gene (ESAG) protein,expression site-associated gene 1 (ESAG1) protein	
Tb09.142.0320	up	hypothetical protein	
Tb09.160.0280	up	variant surface glycoprotein (VSG, atypical), putative	
Tb09.160.1490	up	hypothetical protein, conserved	
Tb09.160.3090	up	heat shock protein, putative,HSP70-like protein	
Tb09.160.3740	up	hypothetical protein, conserved	
Tb09.160.4570	up	arginine kinase	EC:2.7.3.3
Tb09.160.4760	up	hypothetical protein	
Tb09.160.5350	up	variant surface glycoprotein (VSG)-related, putative	
Tb09.160.5380	up	expression site-associated gene 11 (ESAG11) protein, putative	
Tb09.160.5390	up	expression site-associated gene 2 (ESAG2) protein, putative	
Tb09.160.5400	up	expression site-associated gene 9 (ESAG9) protein, putative	
Tb09.v1.0290	up	variant surface glycoprotein (VSG)-related, putative	
Tb09.v1.0300	up	variant surface glycoprotein (VSG)-related, putative	
Tb09.211.0450	up	hypothetical protein, conserved	
Tb09.211.1010	up	phosphatidylcholine:ceramide cholinephosphotransferase 2, putative	EC:2.7.-.-
Tb09.211.1020	up	phosphatidylcholine:ceramide cholinephosphotransferase 2, putative	EC:2.7.-.-
Tb09.v1.0650	up	hypothetical protein, conserved	
Tb09.211.3955	up	hypothetical protein, conserved,predicted heat shock factor binding protein	
Tb09.244.2330	up	variant surface glycoprotein (VSG)-related, putative	
Tb09.244.2190	up	hypothetical protein, conserved	
Tb09.244.2180	up	retrotransposon hot spot (RHS) protein, putative,retrotransposon hot spot protein 3 (RHS3), putative	
Tb09.v4.0010	up	variant surface glycoprotein (VSG), putative,chrIX additional, unordered contigs	
Tb09.v4.0012	up	variant surface glycoprotein (VSG), putative,chrIX additional, unordered contigs	
Tb09.v4.0013	up	retrotransposon hot spot (RHS) protein, putative,chrIX additional, unordered contigs	

Table S2: Robustly regulated transcripts

Tb09.v4.0014	up	hypothetical protein,chrIX additional, unordered contigs	
Tb09.v4.0065	up	expression site-associated gene 1 (ESAG 1), putative,chrIX additional, unordered contigs	
Tb10.70.7150	up	hypothetical protein, conserved	
Tb10.70.7090	up	serine carboxypeptidase III precursor, putative,serine carboxypeptidase (CBP1) precursor, putative,serine peptidase, Clan SC, Family S10	EC:3.4.16.5
Tb10.70.6130	up	hypothetical protein	
Tb10.70.6110	up	hypothetical protein	
Tb10.70.5820	up	hexokinase	EC:2.7.1.1
Tb10.70.5400	up	hypothetical protein, conserved	
Tb10.70.5340	up	protein kinase, putative,casein kinase I, putative	
Tb10.70.5290	up	major surface protease gp63, putative,surface protease homologue	
Tb10.70.5250	up	metacaspase MCA4,cysteine peptidase, Clan CD, family C13, putative	
Tb10.70.5060	up	hypothetical protein, conserved	
Tb10.70.3710	up	aspartate aminotransferase	EC:2.6.1.1
Tb10.70.3610	up	hypothetical protein, conserved	
Tb10.70.3560	up	hypothetical protein, conserved,predicted RING finger protein	
Tb10.70.2990	up	hypothetical protein, conserved	
Tb10.70.2850	up	hypothetical protein	
Tb10.70.2840	up	hypothetical protein	
Tb10.70.2420	up	GPI inositol deacylase precursor	
Tb10.70.2020	up	hypothetical protein, conserved,zinc finger protein family member, putative	
Tb10.70.1850	up	hypothetical protein, conserved,zinc finger protein family member, putative	
Tb10.70.1310	up	procyclin-associated gene 1 (PAG1) protein, putative	
Tb10.70.1300	up	procyclin-associated gene 2 (PAG2) protein, putative	
Tb10.70.1290	up	hypothetical protein, conserved	
Tb10.70.1280	up	hypothetical protein, conserved	
Tb10.70.1130	up	hypothetical protein, conserved	
Tb10.70.0040	up	hypothetical protein, conserved	
Tb10.6k15.3640	up	alternative oxidase	
Tb10.6k15.1970	up	hypothetical protein, conserved	
Tb10.6k15.0940	up	hypothetical protein	
Tb10.6k15.0920	up	expression site-associated gene (ESAG) protein, putative,expression site-associated gene 3 (ESAG3) protein, putative	
Tb10.6k15.0900	up	expression site-associated gene 3 (ESAG3)-like protein	

Table S2: Robustly regulated transcripts

Tb10.6k15.0890	up	expression site-associated gene (ESAG) protein, putative,expression site-associated gene 3 (ESAG3) protein, putative	
Tb10.6k15.0880	up	hypothetical protein	
Tb10.389.1640	up	RNA-binding protein, putative	
Tb10.389.0740	up	hypothetical protein, conserved,zinc finger protein family member, putative	
Tb10.389.0720	up	hypothetical protein, conserved	
Tb10.61.2680	up	pyruvate kinase 1	EC:2.7.1.40
Tb10.61.1750	up	C-terminal motor kinesin, putative	
Tb10.61.1710	up	sarcoplasmic reticulum glycoprotein, putative,sarcalumenin precursor, putative	
Tb10.61.0380	up	glycerol uptake protein, putative	
Tb10.v4.0213	up	expression site-associated gene (ESAG), putative,expression site-associated protein 1 (ESAG1), putative,chrX additional, unordered contig	
Tb10.v4.0217	up	hypothetical protein,chrX additional, unordered contig	
Tb11.47.0026	up	hypothetical protein, conserved	
Tb11.47.0021	up	hypothetical protein, conserved	
Tb11.47.0014	up	hypothetical protein, conserved	
Tb11.47.0001	up	65 kDa invariant surface glycoprotein-like protein	
Tb11.02.0730	up	metacaspase,cysteine peptidase, Clan CD, family C13	
Tb11.02.1564	up	leucine-rich repeat protein (LRRP), putative	
Tb11.02.1565	up	hypothetical protein	
Tb11.02.1566	up	variant surface glycoprotein (VSG)-related, putative	
Tb11.02.1580	up	leucine-rich repeat protein (LRRP), putative	
Tb11.02.1640	up	kinetoplastid-specific dual specificity phosphatase, putative	
Tb11.02.3950	up	ABC transporter, putative	
Tb11.02.4750	up	hypothetical protein, conserved	
Tb11.02.5310	up	major surface protease A, putative,Gp63 major surface glycoprotein-like protein,MSP-A, putative	
Tb11.02.5540	up	hypothetical protein, conserved	
Tb11.02.5610	up	Gp63-1 surface protease homolog, putative	
Tb11.01.0220	up	hypothetical protein, conserved	
Tb11.01.0210	up	hypothetical protein	
Tb11.01.0120	up	haloacid dehalogenase-like hydrolase, putative	
Tb11.01.0090	up	hypothetical protein, conserved,zinc finger protein family member, putative	
Tb11.12.0008	up	hypothetical protein, conserved	
Tb11.12.0016	up	glutathionylspermidine synthetase, putative	
Tb11.01.2460	up	hypothetical protein, conserved	
Tb11.01.2580	up	hypothetical protein, conserved	

Table S2: Robustly regulated transcripts

Tb11.01.3390	up	DNA topoisomerase II, putative	EC:5.99.1.3
Tb11.01.3580	up	hypothetical protein, conserved	
Tb11.01.3940	up	RNA-binding protein, putative	
Tb11.v4.0002	up	hypothetical protein	
Tb11.01.5210	up	hypothetical protein, conserved	
Tb11.01.6010	up	hypothetical protein, conserved	
Tb11.01.6210	up	procyclin-associated gene 2-like protein, putative	
Tb11.01.6220	up	procyclin-associated gene 4 (PAG4) protein, putative	
Tb11.01.6230	up	expression site-associated gene 2 (ESAG2) protein, putative	
Tb11.01.6240	up	expression site-associated gene (ESAG) protein, putative,expression site-associated gene 2 (ESAG2) protein, putative	
Tb11.01.6250	up	expression site-associated gene (ESAG) protein, putative,expression site-associated gene 11 (ESAG11) protein, putative	
Tb11.01.7530	up	hypothetical protein, conserved	
Tb11.17.0002	up	expression site-associated gene (ESAG) protein, putative,expression site-associated gene 1 (ESAG1) protein, putative	
Tb927.1.700	up	phosphoglycerate kinase	EC:2.7.2.3
Tb927.1.1500	up	hypothetical protein, conserved	
Tb927.1.2040	up	expression site-associated gene 2 (ESAG2) protein, putative	
Tb927.1.3550	up	hypothetical protein	
Tb927.1.4490	up	acetyltransferase, putative	
Tb927.1.4600	up	hypothetical protein, conserved	
Tb927.1.4650	up	hypothetical protein, conserved	
Tb927.1.4870	up	expression site-associated gene (ESAG) protein, putative,expression site-associated gene 1 (ESAG1) protein, putative	
Tb927.1.4890	up	expression site-associated gene 2 (ESAG2) protein, putative	
Tb927.1.4900	up	expression site-associated gene 11 (ESAG11) protein, putative	
Tb927.1.4910	up	expression site-associated gene (ESAG) protein, putative,expression site-associated gene 1 (ESAG1) protein, putative	
Tb927.1.5060	up	variant surface glycoprotein (VSG)-related, putative	
Tb927.1.5080	up	expression site-associated gene (ESAG) protein, putative,expression site-associated gene 9 (ESAG9) protein, putative	
Tb927.1.5100	up	expression site-associated gene 2 (ESAG2) protein, putative	
Tb927.1.5110	up	expression site-associated gene 11 (ESAG11) protein, putative	
Tb927.1.5120	up	expression site-associated gene (ESAG) protein, putative,expression site-associated gene 1 (ESAG1) protein, putative	

Table S2: Robustly regulated transcripts

Tb927.1.5170	up	variant surface glycoprotein (VSG)-related, putative	
Tb927.1.5180	up	hypothetical protein	
Tb927.1.5200	up	expression site-associated gene (ESAG) protein, putative,expression site-associated gene 1 (ESAG1) protein, putative	
Tb927.2.2140	down	hypothetical protein, conserved	
Tb927.2.2510	down	hypothetical protein, conserved	
Tb927.2.3610	down	hypothetical protein, conserved	
Tb927.2.4210	down	glycosomal phosphoenolpyruvate carboxykinase,glycosomal protein P60	EC:4.1.1.49
Tb927.2.5700	down	hypothetical protein	
Tb927.3.940	down	hypothetical protein, conserved	
Tb927.3.1380	down	ATP synthase beta chain, mitochondrial precursor,ATP synthase F1, beta subunit	EC:3.6.3.14
Tb927.3.2150	down	protein phosphatase 2C, putative	
Tb927.3.2180	down	hypothetical protein, conserved	
Tb927.3.2230	down	succinyl-CoA synthetase alpha subunit, putative	EC:6.2.1.4
Tb927.3.2650	down	cytochrome c oxidase copper chaperone, putative	
Tb927.3.2880	down	hypothetical protein, conserved	
Tb927.3.3630	down	elongation factor Ts, putative	
Tb927.3.4020	down	phosphatidylinositol 4-kinase alpha, putative	EC:2.7.1.67
Tb927.3.4500	down	fumarate hydratase, putative	EC:4.2.1.2
Tb927.3.4650	down	C-8 sterol isomerase, putative	EC:5.3.3.5
Tb927.3.5780	down	hypothetical protein	
Tb927.4.720	down	hypothetical protein, conserved	
Tb927.4.1360	down	hypothetical protein, conserved	
Tb927.4.3950	down	cytoskeleton-associated protein CAP5.5, putative,cysteine peptidase, Clan CA, family C2, putative	
Tb927.4.4620	down	cytochrome oxidase subunit VIII	
Tb927.4.4730	down	amino acid transporter, putative	
Tb927.4.4990	down	ubiquinol-cytochrome C reductase, putative	EC:1.10.2.2
Tb927.4.5340	down	hypothetical protein, conserved	
Tb927.5.440	down	hypothetical protein, conserved	
Tb927.5.510	down	hypothetical protein, conserved	
Tb927.5.930	down	NADH-dependent fumarate reductase	EC:1.3.1.6
Tb927.5.1060	down	mitochondrial processing peptidase, beta subunit, putative,metallo-peptidase, Clan ME, Family M16	EC:1.10.2.2
Tb927.5.1150	down	pre-mRNA splicing factor ATP-dependent RNA helicase, putative,pre-mRNA splicing factor ATP-dependent RNA helicase, putative	
Tb927.5.2160	down	hypothetical protein, conserved	
Tb927.5.2260	down	hypothetical protein, conserved	
Tb927.5.2560	down	hypothetical protein, conserved	
Tb927.5.2930	down	hypothetical protein, conserved	
Tb927.5.3040	down	hypothetical protein, conserved	
Tb927.5.3090	down	hypothetical protein, conserved	

Table S2: Robustly regulated transcripts

Tb927.5.3340	down	hypothetical protein, conserved	
Tb927.5.3350	down	iron superoxide dismutase, putative	EC:1.15.1.1
Tb927.5.3400	down	calcium-translocating P-type ATPase,calcium pump	EC:3.6.3.6
Tb927.5.4020	down	hypothetical protein	
Tb927.6.510	down	GPEET2 procyclin precursor,PARP A-alpha,procyclin A-alpha,procyclic form specific polypeptide A-alpha precursor	
Tb927.6.520	down	EP3-2 procyclin,PARP A-beta,surface protein EP3-3 procyclin precursor,procyclic form specific polypeptide A-beta precursor	
Tb927.6.2070	down	hypothetical protein, conserved	
Tb927.6.2170	down	co-chaperone GrpE, putative	
Tb927.6.2490	down	hypothetical protein, conserved	
Tb927.6.4130	down	hypothetical protein, conserved	
Tb927.6.4540	down	3-hydroxy-3-methylglutaryl-CoA reductase, putative	EC:1.1.1.34
Tb927.6.4570	down	hypothetical protein, conserved	
Tb927.6.4990	down	ATP synthase, epsilon chain, putative	EC:3.6.3.-
Tb927.7.210	down	proline dehydrogenase	EC:1.5.99.8
Tb927.7.840	down	hypothetical protein, conserved	
Tb927.7.1470	down	ATPase subunit 9, putative	EC:3.6.3.14
Tb927.7.2570	down	hypothetical protein, conserved	
Tb927.7.2640	down	hypothetical protein, conserved	
Tb927.7.2680	down	hypothetical protein, conserved,zinc finger protein family member, putative	
Tb927.7.2700	down	NADH-cytochrome b5 reductase, putative	EC:1.6.2.2
Tb927.7.3020	down	hypothetical protein, conserved	
Tb927.7.3050	down	hypothetical protein, conserved	
Tb927.7.3100	down	hypothetical protein, conserved	
Tb927.7.3280	down	translation initiation factor IF-2, putative	
Tb927.7.4070	down	calpain-like cysteine peptidase, putative,cysteine peptidase, Clan CA, family C2, putative	
Tb927.7.4970	down	glutamine synthetase, putative	EC:6.3.1.2
Tb927.7.6030	down	hypothetical protein	
Tb927.7.6850	down	trans-sialidase	EC:3.2.1.18
Tb927.7.7090	down	hypothetical protein, conserved	
Tb927.7.7420	down	ATP synthase alpha chain, mitochondrial precursor,ATP synthase F1, alpha subunit	
Tb927.7.7470	down	receptor-type adenylate cyclase GRESAG 4, putative	EC:4.6.1.1
Tb927.8.1740	down	hypothetical protein, conserved	
Tb927.8.1790	down	hypothetical protein, conserved	
Tb927.8.1890	down	cytochrome c1, heme protein, mitochondrial precursor	EC:1.10.2.2
Tb927.8.2540	down	3-ketoacyl-CoA thiolase, putative	EC:2.3.1.16
Tb927.8.2770	down	hypothetical protein, conserved	
Tb927.8.4050	down	hypothetical protein, conserved	
Tb927.8.4380	down	hypothetical protein, conserved	
Tb927.8.4630	down	hypothetical protein, conserved	
Tb927.8.4810	down	prohibitin	
Tb927.8.5950	down	protein kinase, putative	EC:2.7.1.-

Table S2: Robustly regulated transcripts

Tb927.8.6110	down	hypothetical protein, conserved	
Tb927.8.6410	down	short-chain dehydrogenase, putative	
Tb927.8.6580	down	succinate dehydrogenase flavoprotein, putative	
Tb927.8.6750	down	translationally controlled tumor protein (TCTP), putative,IgE-dependent histamine-releasing factor, putative	
Tb927.8.7120	down	farnesyltransferase, putative,squalene synthase, putative	EC:2.5.1.21
Tb927.8.7290	down	hypothetical protein, conserved	
Tb927.8.7340	down	trans-sialidase, putative,neuraminidase, putative	
Tb927.8.7350	down	trans-sialidase, putative,neuraminidase, putative	
Tb927.8.7530	down	3,2-trans-enoyl-CoA isomerase, mitochondrial precursor, putative	EC:5.3.3.8
Tb927.8.7540	down	hypothetical protein	
Tb927.8.7730	down	hypothetical protein, conserved	
Tb927.8.8300	down	amino acid transporter, putative	
Tb09.160.0465	down	hypothetical protein, conserved	
Tb09.160.0510	down	hypothetical protein, conserved	
Tb09.160.1100	down	hypothetical protein, conserved	
Tb09.160.1820	down	cytochrome oxidase subunit V	
Tb09.160.2160	down	hypothetical protein, conserved	
Tb09.160.4950	down	hypothetical protein, conserved	
Tb09.160.5060	down	hypothetical protein, conserved	
Tb09.160.5260	down	oxidoreductase, putative	EC:1.6.5.5
Tb09.v1.0420	down	hypothetical protein, conserved	
Tb09.211.0040	down	hypothetical protein, conserved	
Tb09.211.1240	down	hypothetical protein, conserved	
Tb09.211.1800	down	hypothetical protein, conserved	
Tb09.211.1940	down	hypothetical protein, conserved	
Tb09.211.3330	down	cystathione gamma lyase, putative	EC:4.4.1.1
Tb09.211.3680	down	chaperone protein DNAJ, putative	
Tb09.211.4070	down	hypothetical protein	
Tb09.244.2700	down	hypothetical protein, conserved	

Table S2: Robustly regulated transcripts

Tb10.100.0070	down	ATP synthase F1 subunit gamma protein, putative	EC:3.6.3.14
Tb10.100.0160	down	cytochrome oxidase subunit VI	
Tb10.70.6660	down	hypoxanthine-guanine phosphoribosyltransferase, putative	
Tb10.70.6470	down	methionyl-tRNA synthetase, putative	EC:6.1.1.10
Tb10.70.6340	down	ATPase subunit 9, putative	EC:3.6.3.14
Tb10.70.5110	down	mitochondrial malate dehydrogenase	EC:1.1.1.37
Tb10.70.4590	down	hypothetical protein, conserved	
Tb10.70.4140	down	hypothetical protein, conserved	
Tb10.70.3010	down	hypothetical protein, conserved	
Tb10.70.2970	down	hypothetical protein, conserved	
Tb10.70.2320	down	hypothetical protein, conserved	
Tb10.70.2155	down	hypothetical protein, conserved	
Tb10.70.0625	down	hypothetical protein, conserved	
Tb10.6k15.3510	down	cysteine-rich, acidic integral membrane protein precursor	
Tb10.6k15.2510	down	hypothetical protein, conserved	
Tb10.6k15.2180	down	cytochrome oxidase subunit IX	
Tb10.6k15.1350	down	pteridine transporter, putative	
Tb10.6k15.0500	down	hypothetical protein, conserved	
Tb10.6k15.0280	down	hypothetical protein, conserved	
Tb10.6k15.0030	down	EP2 procyclin	
Tb10.389.1480	down	cytosolic nonspecific dipeptidase, putative,peptidase (M20/M25/M40 family), putative	
Tb10.389.1180	down	P-type H+-ATPase, putative	EC:3.6.3.6
Tb10.389.0910	down	60S ribosomal protein L34, putative	
Tb10.389.0770	down	hypothetical protein, conserved,zinc finger protein family member, putative	
Tb10.389.0690	down	mitochondrial carrier protein, putative,mitochondrial 2-oxoglutarate/malate carrier protein, putative	
Tb10.389.0070	down	elongation factor TU, putative	
Tb10.61.2745	down	mevalonate diphosphate decarboxylase	EC:4.1.1.33
Tb10.61.0980	down	glycosomal malate dehydrogenase	EC:1.1.1.37
Tb10.61.0395	down	hypothetical protein, conserved	
Tb10.61.0370	down	hypothetical protein, conserved	
Tb10.61.0220	down	hypothetical protein, conserved	
Tb10.61.0200	down	hypothetical protein, conserved	
Tb11.47.0022	down	hypothetical protein, conserved	

Table S2: Robustly regulated transcripts

Tb11.47.0004	down	2-oxoglutarate dehydrogenase E1 component, putative	
Tb11.22.0001	down	hypothetical protein, conserved	
Tb11.02.0445	down	hypothetical protein, conserved	
Tb11.02.0580	down	vesicular protein trafficking mediator, putative	
Tb11.02.1480	down	mitochondrial processing peptidase alpha subunit, putative,metallo-peptidase, Clan ME, Family M16	EC:3.4.99.41
Tb11.02.2310	down	prostaglandin f synthase	EC:1.1.-.-
Tb11.02.2950	down	ATPase subunit 9, putative	EC:3.6.3.14
Tb11.02.3120	down	malic enzyme, putative	EC:1.1.1.38
Tb11.02.3180	down	hypothetical protein, conserved	
Tb11.02.3210	down	triosephosphate isomerase	EC:5.3.1.1
Tb11.02.4080	down	lanosterol 14-alpha-demethylase,cytochrome P450 51A1	EC:1.14.13.70
Tb11.02.4120	down	hypothetical protein, conserved	
Tb11.02.4150	down	pyruvate phosphate dikinase	EC:2.7.9.1
Tb11.02.4910	down	acidocalcisomal pyrophosphatase	
Tb11.02.5160	down	hypothetical protein, conserved	
Tb11.02.5165	down	hypothetical protein, conserved	
Tb11.02.5400	down	cystathionine beta-synthase, putative	EC:4.2.1.22
Tb11.02.5480	down	hypothetical protein, conserved	
Tb11.02.5490	down	hypothetical protein, conserved	
Tb11.02.5590	down	hypothetical protein, conserved	
Tb11.02.5660	down	hypothetical protein, conserved	
Tb11.02.5770	down	mitochondrial RNA binding protein,RBP16,RNA-binding protein of 16 kDa	
Tb11.01.0720	down	cation transporter, putative	
Tb11.01.1480	down	60S ribosomal protein L34, putative	
Tb11.01.6410	down	phosphomannose isomerase, putative	EC:5.3.1.8
Tb11.01.6660	down	iron superoxide dismutase	EC:1.15.1.1
Tb11.01.7170	down	C-14 sterol reductase, putative	
Tb11.01.7880	down	microtubule-associated protein,corset-associated protein 17	
Tb11.01.8225	down	hypothetical protein, conserved	
Tb11.01.8470	down	dihydrolipoyl dehydrogenase	EC:1.8.1.4
Tb927.1.710	down	phosphoglycerate kinase	EC:2.7.2.3
Tb927.1.2230	down	calpain-like protein fragment, putative	
Tb927.1.2260	down	calpain-like protein fragment, putative	
Tb927.1.3310	down	hypothetical protein, conserved	
