# Supplementary material for: Spliced Leader Trapping Reveals Widespread Alternative Splicing Patterns in the Highly Dynamic Transcriptome of Trypanosoma brucei
Source: PLoS Pathog. 2010 Aug 5;6(8):e1001037. doi: 10.1371/journal.ppat.1001037 (PMC2916883; doi:10.1371/journal.ppat.1001037)
Supplement: Table S3 — KEGG pathways and their expression levels in three life cycle stage (0.08 MB PDF) [file ppat.1001037.s016.pdf]

Table S3: *T. bruei* annotated KEGG pathways and their expression levels in three life cycle stages

Pathway	Name	LongSlender	ShortStumpy	PC	LS/SS	PC/LS	PC/SS
500	Starch and sucrose metabolism	444	49	118	9.06	0.27	2.41
52	Galactose metabolism	435	65	122	6.69	0.28	1.88
592	alpha-Linolenic acid metabolism	12	2	64	6	5.33	32.00
750	Vitamin B6 metabolism	89	21	51	4.24	0.57	2.43
565	Ether lipid metabolism	108	40	89	2.7	0.82	2.23
330	Arginine and proline metabolism	2291	865	1920	2.65	0.84	2.22
10	Glycolysis / Gluconeogenesis	3620	1420	5089	2.55	1.41	3.58
760	Nicotinate and nicotinamide metabolism	270	110	138	2.45	0.51	1.25
642	Ethylbenzene degradation	18	9	94	2	5.22	10.44
51	Fructose and mannose metabolism	1292	660	2047	1.96	1.58	3.10
630	Glyoxylate and dicarboxylate metabolism	357	193	757	1.85	2.12	3.92
780	Biotin metabolism	107	62	407	1.73	3.8	6.56
620	Pyruvate metabolism	1637	978	2393	1.67	1.46	2.45
3050	Proteasome	1939	1190	2235	1.63	1.15	1.88
272	Cysteine metabolism	437	269	75	1.62	0.17	0.28
360	Phenylalanine metabolism	431	268	84	1.61	0.19	0.31
72	Synthesis and degradation of ketone bodies	64	41	144	1.56	2.25	3.51
983	Drug metabolism - other enzymes	234	153	266	1.53	1.14	1.74
590	Arachidonic acid metabolism	26	17	9	1.53	0.35	0.53
480	Glutathione metabolism	2434	1622	2389	1.5	0.98	1.47
4020	Calcium signaling pathway	1104	764	1297	1.45	1.17	1.70
61	Fatty acid biosynthesis	402	277	319	1.45	0.79	1.15
530	Aminosugars metabolism	1331	927	802	1.44	0.6	0.87
564	Glycerophospholipid metabolism	585	408	926	1.43	1.58	2.27
251	Glutamate metabolism	1315	928	1173	1.42	0.89	1.26
252	Alanine and aspartate metabolism	1579	1178	1243	1.34	0.79	1.06
720	Reductive carboxylate cycle (CO2 fixation)	510	383	1157	1.33	2.27	3.02
790	Folate biosynthesis	411	312	292	1.32	0.71	0.94
3060	Protein export	305	233	615	1.31	2.02	2.64
350	Tyrosine metabolism	561	430	167	1.3	0.3	0.39
400	Phenylalanine, tyrosine and tryptophan biosynthesis	517	401	210	1.29	0.41	0.52
460	Cyanoamino acid metabolism	968	765	601	1.27	0.62	0.79
970	Aminoacyl-tRNA biosynthesis	1328	1055	1965	1.26	1.48	1.86
230	Purine metabolism	8028	6375	7099	1.26	0.88	1.11
910	Nitrogen metabolism	1599	1300	812	1.23	0.51	0.62
3440	Homologous recombination	791	646	623	1.22	0.79	0.96
900	Terpenoid biosynthesis	164	139	414	1.18	2.52	2.98
4120	Ubiquitin mediated proteolysis	1367	1173	1230	1.17	0.9	1.05
632	Benzoate degradation via CoA ligation	295	256	480	1.15	1.63	1.88
511	Other glycan degradation	279	242	307	1.15	1.1	1.27
3030	DNA replication	1692	1476	1181	1.15	0.7	0.80
600	Sphingolipid metabolism	381	333	515	1.14	1.35	1.55
71	Fatty acid metabolism	1194	1059	1970	1.13	1.65	1.86
563	Glycosylphosphatidylinositol(GPI)-anchor biosynthesis	131	116	139	1.13	1.06	1.20
53	Ascorbate and aldarate metabolism	19	17	131	1.12	6.89	7.71
1040	Biosynthesis of unsaturated fatty acids	511	458	1417	1.12	2.77	3.09
770	Pantothenate and CoA biosynthesis	153	136	205	1.12	1.34	1.51
3420	Nucleotide excision repair	1733	1547	1169	1.12	0.67	0.76
130	Ubiquinone and menaquinone biosynthesis	42	38	55	1.11	1.31	1.45
3430	Mismatch repair	1458	1310	1104	1.11	0.76	0.84
640	Propanoate metabolism	5043	4556	4611	1.11	0.91	1.01
290	Valine, leucine and isoleucine biosynthesis	187	178	372	1.05	1.99	2.09
3410	Base excision repair	1120	1069	692	1.05	0.62	0.65
240	Pyrimidine metabolism	2468	2373	5004	1.04	2.03	2.11
2010	ABC transporters	52	50	18	1.04	0.35	0.36
20	Citrate cycle (TCA cycle)	6568	6354	6712	1.03	1.02	1.06
120	Bile acid biosynthesis	107	105	255	1.02	2.38	2.43
190	Oxidative phosphorylation	6397	6287	9436	1.02	1.48	1.50
4070	Phosphatidylinositol signaling system	569	558	562	1.02	0.99	1.01
510	N-Glycan biosynthesis	407	401	325	1.01	0.8	0.81
440	Aminophosphonate metabolism	195	196	197	0.99	1.01	1.01
930	Caprolactam degradation	164	170	397	0.96	2.42	2.34
670	One carbon pool by folate	77	80	75	0.96	0.97	0.94
280	Valine, leucine and isoleucine degradation	677	710	1203	0.95	1.78	1.69
3020	RNA polymerase	971	1023	989	0.95	1.02	0.97
650	Butanoate metabolism	294	313	718	0.94	2.44	2.29
410	beta-Alanine metabolism	194	210	534	0.92	2.75	2.54
281	Geraniol degradation	248	269	559	0.92	2.25	2.08
380	Tryptophan metabolism	1557	1695	1865	0.92	1.2	1.10
62	Fatty acid elongation in mitochondria	23	25	23	0.92	1	0.92
641	3-Chloroacrylic acid degradation	10	11	116	0.91	11.6	10.55
561	Glycerolipid metabolism	263	303	548	0.87	2.08	1.81
562	Inositol phosphate metabolism	508	582	632	0.87	1.24	1.09
310	Lysine degradation	1391	1600	1930	0.87	1.39	1.21
903	Limonene and pinene degradation	219	257	614	0.85	2.8	2.39
220	Urea cycle and metabolism of amino groups	1786	2096	2052	0.85	1.15	0.98
150	Androgen and estrogen metabolism	148	177	95	0.84	0.64	0.54
3450	Non-homologous end-joining	290	351	247	0.83	0.85	0.70
785	Lipoic acid metabolism	607	733	367	0.83	0.6	0.50
624	1- and 2-Methylnaphthalene degradation	91	111	153	0.82	1.68	1.38
340	Histidine metabolism	143	174	202	0.82	1.41	1.16
860	Porphyrin and chlorophyll metabolism	330	401	220	0.82	0.67	0.55
30	Pentose phosphate pathway	3272	4026	2936	0.81	0.9	0.73

Table S3: *T. bruei* annotated KEGG pathways and their expression levels in three life cycle stages

791	Atrazine degradation	36	45	94	0.8	2.61	2.09
271	Methionine metabolism	3612	4488	4061	0.8	1.12	0.90
450	Selenoamino acid metabolism	2010	2587	2498	0.78	1.24	0.97
4140	Regulation of autophagy	651	841	304	0.77	0.47	0.36
626	Naphthalene and anthracene degradation	198	262	188	0.76	0.95	0.72
4130	SNARE interactions in vesicular transport	869	1155	816	0.75	0.94	0.71
363	Bisphenol A degradation	51	70	54	0.73	1.06	0.77
550	Peptidoglycan biosynthesis	74	102	78	0.73	1.05	0.76
361	gamma-Hexachlorocyclohexane degradation	14	20	46	0.7	3.29	2.30
3010	Ribosome	250198	357980	341987	0.7	1.37	0.96
40	Pentose and glucuronate interconversions	103	159	84	0.65	0.82	0.53
260	Glycine, serine and threonine metabolism	953	1500	1747	0.64	1.83	1.16
520	Nucleotide sugars metabolism	24	38	32	0.63	1.33	0.84
3022	Basal transcription factors	133	210	29	0.63	0.22	0.14
100	Biosynthesis of steroids	826	1326	1158	0.62	1.4	0.87
