# Supplementary material for: Spliced Leader Trapping Reveals Widespread Alternative Splicing Patterns in the Highly Dynamic Transcriptome of Trypanosoma brucei
Source: PLoS Pathog. 2010 Aug 5;6(8):e1001037. doi: 10.1371/journal.ppat.1001037 (PMC2916883; doi:10.1371/journal.ppat.1001037)
Supplement: Table S4 — Correlation of expression profile between SLT and qPCR for 10 selected genes (0.03 MB PDF) [file ppat.1001037.s017.pdf]

Table S4: Correlation of expression profile between SLT and qPCR for 10 selected genes

**ID**	**log_2 SLT changes procyclics/long slender**	**log_2 qPCR changes procyclics/long slender**
Tb927.7.3730	-2.77	-1.77
Tb927.7.4230	-2.37	-2.43
Tb10.70.3610	-3.79	-2.67
Tb10.61.0370	1.15	0.50
Tb11.02.5490	5.18	3.24
Tb11.02.5500	-0.51	-0.70
Tb10.389.0080	1.36	-0.88
Tb927.7.4650	-3.09	-1.79
Tb927.6.510	5.15	2.74
Tb10.6k15.3640	-4.61	-2.84
