# Supplementary material for: Spliced Leader Trapping Reveals Widespread Alternative Splicing Patterns in the Highly Dynamic Transcriptome of Trypanosoma brucei
Source: PLoS Pathog. 2010 Aug 5;6(8):e1001037. doi: 10.1371/journal.ppat.1001037 (PMC2916883; doi:10.1371/journal.ppat.1001037)
Supplement: Table S5 — Proteins with N-terminal extensions (0.04 MB PDF) [file ppat.1001037.s018.pdf]

Table S5: Proteins with N-terminal extensions

ID	Position of peptide relative to anotated AUG in nucleotides	Position of peptide relative to anotated AUG in amino acids	Total number of tags	Tags per million (TPM)	Number of tags/splice site	Length of UTRs	Length of major UTR	TPM of major UTR	Description
Tb927.3.5490	-12	-4	112	96	98,5,9	267,247,110	267	98	flagellar transport protein
Tb927.5.3170	-546	-182	97	83	11,12,1,73	1080,1085,1087,1090	1090	73	ribose-phosphate pyrophosphokinase
Tb927.7.1550	-81	-27	38	32	1,34,1,2	438,329,323,232	329	34	terminal uridylyl transferase, KRET2
Tb927.7.3280	-66	-22	129	110	1,1,123,4	238,240,256,737	256	123	translation initiation factor IF-2 putative
Tb927.7.7440	-39	-13	29	25	3,9,2,15	175,205,1832,1836	1836	15	hypothetical protein conserved
Tb09.211.0330	-177	-59	100	85	2,10,54,34	574,582,593,596	593	54	chaperone protein DNAJ
Tb10.70.7880	-69	-23	30	26	25,4,1	469,464,444	469	25	hypothetical protein conserved
Tb10.70.3980	-63	-21	1	1	1	-1266	-1266	1	hypothetical protein conserved
Tb10.61.0150	-36	-12	124	106	120,4	449,-185	449	120	IMP dehydrogenase
Tb11.02.2740	-30	-10	30	26	26,3,1	201,196,161	201	26	aspartate aminotransferase mitochondrial
Tb11.01.8510	-75	-25	258	220	242,4,12	170,193,782	170	242	t-complex protein
