# Supplementary material for: Spliced Leader Trapping Reveals Widespread Alternative Splicing Patterns in the Highly Dynamic Transcriptome of Trypanosoma brucei
Source: PLoS Pathog. 2010 Aug 5;6(8):e1001037. doi: 10.1371/journal.ppat.1001037 (PMC2916883; doi:10.1371/journal.ppat.1001037)
Supplement: Table S6 — Comparison of expression levels from differential splice sites using RT qPCR and SLT (0.04 MB PDF) [file ppat.1001037.s019.pdf]

Table S6: Comparison of expression levels from differential splice sites using RT qPCR and SLT

ID	Long slender/procyclics qPCR	ci min (95%) qPCR	ci max (95%) qPCR	Long slender/procyclics SLT	Pearson
Tb927.1.790up	1.03	0.84	1.26	2.11	0.85
Tb927.1.790down	2.69	2.26	3.20	2.80	
Tb11.02.2700up	1.37	0.93	2.02	1.00	
Tb11.02.2700down	2.66	1.36	5.23	1.47	
Tb927.6.4240up	1.29	0.94	1.78	1.00	
Tb927.6.4240down	5.50	3.87	7.82	4.05	

ci 95% confidence interval

up and down refers to the up- and downstream splice sites
