# Supplementary material for: Spliced Leader Trapping Reveals Widespread Alternative Splicing Patterns in the Highly Dynamic Transcriptome of Trypanosoma brucei
Source: PLoS Pathog. 2010 Aug 5;6(8):e1001037. doi: 10.1371/journal.ppat.1001037 (PMC2916883; doi:10.1371/journal.ppat.1001037)
Supplement: Table S7 — Alternative splicing and dual localization of tRNA Synthetases (0.07 MB PDF) [file ppat.1001037.s020.pdf]

Table S7: Alternative splicing and dual localization of tRNA Synthetases

Gene ID	Name [1]	MTS (p) [2]	MTS (aa) [3]	TPM [4]	tN-term (aa) [5]	iMTS (p) [6]	iMTS (aa) [7]
**Aminoacyl tRNA Synthetases (RS) with two genome-encoded isoforms**							
Tb10.70.6800	aspartyl-RS, **M**	0.91	72	107	-	-	-
Tb927.6.1880	aspartyl-RS, **C**	0.02	np	11	-	-	-
Tb927.6.1510	lysyl-RS, **M**	0.99	28	61	-	-	-
Tb927.8.1600	lysyl-RS, **C**	0.23	np	225	-	-	-
Tb927.8.2240	tryptophanyl-RS, **M**	0.26	28	1	-	-	-
Tb927.3.5580	tryptophanyl-RS, **C**	0.08	np	55	-	-	-
Tb927.7.2400	tyrosyl-RS, **M**	0.64	np	1	-	-	-
Tb927.7.3620	tyrosyl-RS, **C**	0.0277	np	24	-	-	-
**Aminoacyl tRNA Synthetases (RS) where alternative splicing could lead to dual localization**							
Tb927.4.2310	asparaginyl-RS,	0.95	49	62	-	-	-
Tb10.389.0630	prolyl-RS,	0.98	102	145	-	-	-
Tb09.160.3730	glutaminyl-RS,	0.96	30	295	-	-	-
Tb927.6.4590	glutamyl-RS,	0.91	20	13	-	-	-
Tb10.6k15.1220	isoleucyl-RS,	0.81	43	120	-	-	-
**Aminoacyl tRNA Synthetases (RS) with one isoform with potential internal MTS (iMTS)**							
Tb11.46.0008	arginyl-RS,	0.12	np	155	101	0.87	np
Tb927.6.950	cysteinyl-RS,	0.01	np	1	73	0.71	np
Tb927.6.2060	histidyl-RS,	0.14	np	2	48	0.82	49
Tb11.02.5020	leucyl-RS,	0.07	np	20	49	0.90	26
**Aminoacyl tRNA Synthetases (RS) with unknown localization mechanisms**							
Tb927.6.700	alanyl-RS,	0.20	np	69	-	-	-
Tb927.8.5330	tyrosyl/methionyl-RS,	0.27	np	216	-	-	-
Tb10.70.6470	methionyl-RS,	0.37	np	45	-	-	-
Tb11.22.0005	phenylalanyl-RS (beta)	0.27	np	3	-	-	-
Tb11.01.5710	phenylalanyl-RS (alpha)	0.45	np	122	-	-	-
Tb11.01.1400	glycyl-RS,	0.24	np	3	-	-	-
Tb927.5.1090	threonyl-RS,	0.03	np	27	-	-	-
Tb927.6.4480	valyl-RS,	0.12	np	29	-	-	-
Tb11.02.5020	seryl-RS,	0.05	np	20	-	-	-

[1] Name of tRNA S., M represents the mitochondrial isoform, C represents the cytosolic isoform
[2] Mitochondrial targeting signal prediction probability as determined using MITOPROT.
[3] Length of the mitochondrial targeting signal determined using MITOPROT; np: not predictable.
[4] Tags per million (TPM) of the major splice site as determined by SLT.
[5] N-terminal truncation (tN-term) by internal splice site, length of the truncation in amino acids (aa)
[6] Mitochondrial targeting signal prediction probability of the internal targeting sequence as determined using MITOPROT.
[7] Length of the internal mitochondrial targeting signal as determined using MITOPROT; np: not predictable.
