# Supplementary material for: Spliced Leader Trapping Reveals Widespread Alternative Splicing Patterns in the Highly Dynamic Transcriptome of Trypanosoma brucei
Source: PLoS Pathog. 2010 Aug 5;6(8):e1001037. doi: 10.1371/journal.ppat.1001037 (PMC2916883; doi:10.1371/journal.ppat.1001037)
Supplement: Table S8 — Oligonucleotide sequences (0.04 MB PDF) [file ppat.1001037.s021.pdf]

Table S8: RT qPCR oligonucleotides

GeneID	Oligonucleotide sequence
**qPCR differential splicing**	
tb927.6.4240upF	TTCTTCTCAGCTTGCTGTCC
tb927.6.4240upR	TTTACTGCTGCGATGGTTTC
tb927.6.4240downF	CTCCCAATATCCTTTGCCAT
tb927.6.4240downR	TCACCGAATTCACTGCAAAT
tb11.02.2700upF	CAACAACAACAGCAACATCAA
tb11.02.2700upR	GACTTGGGTGCAAACCAGA
tb11.02.2700downF	CCGGAAATTCTAAAGGTGGA
tb11.02.2700downR	TTCTTAAGCTCGCCAGGTG
tb927.1.790upF	TTCAGCTGAGACGATAACCG
tb927.1.790upR	TGGTGTTAGTTCCTCGCTTG
tb927.1.790downF	AACTTTGCAATTTCCCGAAG
tb927.1.790downR	GGTCCTGTTTCTAGGCTTGG
**qPCR gene expression levels**	
Tb927.7.3730f	ATGCATACGACCTCTGTCCA
Tb927.7.3730r	TCCTTGACTACCTCGAGCCT
Tb927.7.4230f	TCATAACGCGTAAATGGGAA
Tb927.7.4230r	CCTCTGTTCTTGTTCTGGCA
Tb10.70.3610f	CAACATAATGCGGAATAGCG
Tb10.70.3610r	CCGACATTGCATTTGGATAG
Tb10.61.0370f	AGCACTGCGTATCCTTCCTT
Tb10.61.0370r	AAGTGCAACATGCCGAGTTA
Tb11.02.5490f	AAGGTTAGGGTCCAGCCAGT
Tb11.02.5490r	CACTACAACCATGGGCAAAG
Tb11.02.5500f	ACGCAGGTACCGTTACAACA
Tb11.02.5500r	GTGCTGCAGAAGATGAAGGA
Tb10.389.0080f	TAGAGGGATTCGTGGTGTGA
Tb10.389.0080r	CAAAGCGATTCGTTTCAAGA
Tb927.7.4650f	ACCTTGCCTGTAGGGATGTC
Tb927.7.4650r	AAGCAGCTGTAAACATTGCG
Tb927.6.510f	GTTGCCAATGAAGGTGACAG
Tb927.6.510r	GTTCCCTTTATCTGCTCGCT
Tb10.6k15.3640f	TCCTCCCTCTCCTTTCCTT
Tb10.6k15.3640r	TACGCACAGAAGCCAAAC
